# Congo Red–Functionalized Maize Stalk for Fe^3+^, Cr^3+^ and Mn^2+^ Adsorption: Multi-Analytical Characterization of Interaction Mechanisms

**DOI:** 10.3390/polym18131600

**Published:** 2026-06-27

**Authors:** Nicoleta Mirela Marin, Toma Galaon, Adriana Mariana Borș, Roxana Doina Trusca, Ludmila Motelica, Ovidiu Oprea

**Affiliations:** 1 National Research and Development Institute for Industrial Ecology ECOIND, Street Podu Dambovitei No. 57-73, District 6, 060652 Bucharest, Romania; tomagalaon@yahoo.com; 2 Department of Analytical and Physical Chemistry, University of Bucharest, 4-12 Regina Elisabeta Bd., 030018 Bucharest, Romania; 3 Department of Oxide Materials Science and Engineering, National University of Science and Technology POLITEHNICA Bucharest, 1–7 Gh. Polizu, 060042 Bucharest, Romania; 4 National Institute for R&D for Optoelectronics-Subsidiary, Research Institute for Hydraulics and Pneumatics—INOE 2000-IHP, 040558 Bucharest, Romania; bors.ihp@fluidas.ro; 5 National Centre for Micro- and Nanomaterials, National University of Science and Technology POLITEHNICA Bucharest, 313 Independence Boulevard, 060042 Bucharest, Romania; truscaroxana@yahoo.com (R.D.T.); ludmila.motelica@upb.ro (L.M.); ovidiu.oprea@upb.ro (O.O.); 6 Research Center for Advanced Materials, Products and Processes, National University of Science and Technology POLITEHNICA Bucharest, Splaiul Independenței 313, 060042 Bucharest, Romania; 7 Academy of Romanian Scientists, 3 Ilfov St., 050045 Bucharest, Romania; 8 Faculty of Chemical Engineering and Biotechnologies, National University of Science and Technology POLITEHNICA Bucharest, Gh. Polizu 1-7, 011061 Bucharest, Romania

**Keywords:** maize stalk, Congo red, metal ions, adsorption, coordination, equilibrium

## Abstract

This study examines the adsorption and interaction mechanisms of Congo red (CR) immobilized onto maize stalk (MS) to form MS-CR material, used for the removal of Fe^3+^, Cr^3+^, and Mn^2+^ (M^n+^) from aqueous media. Initially, the MS was functionalized with CR, achieving adsorption capacities between 41.4 and 48.0 mg/g across pH 2–10, confirming the formation of hydrogen bonding and aromatic interactions, as indicated by the shift of the OH band from 3338.91 to 3335.54 cm^−1^ and the appearance of characteristic azo–aromatic peaks (1601–1506 cm^−1^) in the FTIR spectra. Stability tests showed that CR remains anchored to the lignocellulosic matrix even under 2 M HCl/NaOH. Subsequently, adsorption experiments revealed a strong pH dependence: at pH 10, removal efficiencies reached 93% for Mn^2+^, 89% for Fe^3+^, and 72% for Cr^3+^ at 2 mg/L, driven by surface deprotonation and enhanced electrostatic attraction. Increasing the initial metal concentration (1–10 mg/L) led to maximum adsorption capacities of 2.00 mg/g for Fe^3+^, 1.64 mg/g for Cr^3+^, and 1.46 mg/g for Mn^2+^. Desorption experiments identified 0.5 M HCl as the optimal regenerating agent, achieving 90–97% metal release. FTIR analysis of MS-CR–Mn^2+^ showed the disappearance of the 1243 cm^−1^ carboxyl band and the emergence of a metal–oxygen vibration at 559.37 cm^−1^, confirming adsorption via coordination to deprotonated carboxyl and phenolic groups. TG/DSC/DTG analysis demonstrated improved stability of MS-CR compared to native MS. SEM/EDX confirmed the presence of S, Na, and M^n+^. The combined spectroscopic, microscopic, and thermal evidence demonstrates that MS-CR operates as a robust, multifunctional adsorbent capable of M^n+^ retention, offering a sustainable solution for water treatment.

## 1. Introduction

Today, the increasing presence of dissolved metals in natural and industrial effluents poses significant environmental and public-health risks, driving the need for efficient, low-cost, and sustainable remediation technologies [1,2,3,4,5].

Iron (Fe), chromium (Cr), and manganese (Mn) are essential trace elements, yet their dissolved ionic forms—particularly Fe^3+^, Cr^3+^, and Mn^2+^—can become hazardous when present at elevated concentrations in aquatic systems due to industrial discharge, mining effluents, corrosion of metal infrastructure, and leaching from contaminated soils [6,7,8].

Although Fe^3+^ is required for oxygen transport and enzymatic activity, excess levels promote oxidative stress, gastrointestinal disorders, and organ damage through the Fenton reaction, which generates reactive oxygen species [9]. Cr^3+^, traditionally considered less toxic than Cr^6+^, can still induce DNA–protein crosslinking, oxidative imbalance, and cytotoxicity at high concentrations, especially in populations exposed to tannery wastewaters, electroplating residues, and metal-finishing industries [10,11]. Mn^2+^, an essential cofactor in metabolic pathways, becomes neurotoxic upon chronic exposure, accumulating in the basal ganglia and leading to manganism—a Parkinson-like syndrome characterized by motor dysfunction, cognitive impairment, and behavioral changes [12,13,14].

Regulatory limits reflect these risks: the WHO guideline values for drinking water are 0.3 mg/L for Fe, 0.05 mg/L for Mn, and 0.5 mg/L for total chromium (with many jurisdictions adopting even stricter thresholds) [15]. Given their persistence, mobility, and capacity to induce oxidative stress, neurotoxicity, and genotoxic effects, the removal of Fe^3+^, Cr^3+^, and Mn^2+^ from contaminated waters remains a critical priority for protecting environmental and public health. In this regard, identifying highly effective materials and treatment processes has become a strategic priority for the protection of ecosystems and public health.

Conventional treatment methods often suffer from high operational costs, limited selectivity, or poor regeneration capacity, underscoring the importance of developing renewable biosorbents capable of delivering high performance under variable water-quality conditions [16,17,18,19,20]. Lignocellulosic agricultural residues represent an abundant and biodegradable resource that can be chemically tailored to enhance their affinity toward metal ions [21,22,23,24,25]. In this context, the functionalization of maize stalk with CR introduces sulfonate, azo, and aromatic groups that can promote electrostatic and coordination interactions with multivalent metals.

This study aimed to develop and validate a renewable and biodegradable MS–CR functionalized polymer. The functionalization of MS with CR and its specific coordination behavior toward Fe^3+^, Cr^3+^, and Mn^2+^ has not yet been addressed in the literature to the best of our knowledge. The involvement of the sulfonate and azo groups of CR in metal binding on a maize-based matrix remains insufficiently elucidated, and a multi-analytical validation of these interactions has likewise been lacking. This work fills this gap by introducing the first MS-CR material designed for Fe^3+^, Cr^3+^, and Mn^2+^ removal and by elucidating its coordination mechanisms through FTIR-ATR, SEM/EDX, TG-DSC/DTG, adsorption, and desorption analyses. The study establishes a distinct functionalization route and demonstrates a stable, efficient biosorbent for the retention of multivalent metals.

## 2. Materials and Methods

### 2.1. Chemicals

CR (disodium 3,3′-[(1,1′-biphenyl)-4,4′-diylbis(azo)]bis(4-aminonaphthalene-1-sulfonate), analytical grade, was purchased from Sigma-Aldrich (St. Louis, MO, USA) and used as received.

Maize stalk (MS) biomass was collected from local agricultural residues (Romania), thoroughly washed with deionized water, dried, ground, and sieved to the desired particle size prior to use. Certified mono-element standard solutions of Fe^3+^, Cr^3+^ and Mn^2+^ (1000 mg/L as nitrate salts, Fe(NO_3_)_3_, Cr(NO_3_)_3_, Mn(NO_3_)_2_; Merck, Darmstadt, Germany) were used to prepare working metal ion solutions by appropriate dilution with deionized water. Hydrochloric acid (HCl, 37%) and sodium hydroxide (NaOH, 50%) (Merck) were employed for tested functionalized maize stability. Acetate, phosphate and carbonate buffer solutions were prepared from analytical-grade reagents (Merck) to obtain stable pH values of 2.0, 4.0, 6.0, 8.0 and 10.0.

### 2.2. FTIR-ATR Characterization

FTIR spectra were recorded at room temperature using a Nicolet iS50R spectrometer (Thermo Fisher Scientific Inc., Madison, WI, USA) equipped with an attenuated total reflectance (ATR) module. The MS, MS–CR, and MS–CR–M^n+^ samples were placed directly onto the diamond ATR crystal to ensure optimal contact during measurement. For each spectrum, 32 scans were collected in the 4000–400 cm^−1^ range with a spectral resolution of 4 cm^−1^. The obtained spectra were used to identify functional groups involved in CR adsorption and metal ion coordination, as well as to compare structural changes between the native, binary, and ternary systems.

### 2.3. SEM/EDX Characterization

Morphological and elemental analyses of MS, MS–CR, and MS–CR–M^n+^ were performed using a Quanta Inspect F50 field-emission scanning electron microscope (FEG SEM) (FEI, Hillsboro, OR, USA), operating at a resolution of 1.2 nm. The instrument is equipped with an energy dispersive X-ray (EDX) detector with a resolution of 133 eV for the MnKα line. SEM micrographs were used to evaluate surface texture modifications induced by CR functionalization and metal adsorption, while EDX spectra confirmed the presence and distribution of Fe^3+^, Cr^3+^, and Mn^2+^ on the MS-CR surface.

### 2.4. TG-DSC Characterization

Thermal behavior of MS, CR, and MS–CR samples was investigated using a Netzsch STA 449C Jupiter simultaneous TG DSC analyzer (NETZSCH Gerätebau GmbH, Selb, Germany). Approximately 10–12 mg of sample was placed in alumina crucibles with pierced lids and heated from room temperature to 900 °C at a rate of 10 K/min, under a continuous flow of 50 mL/min dry air. An empty alumina crucible served as a reference. The TG DSC curves were used to assess thermal stability, decomposition stages, and potential interactions between the biomass matrix and the adsorbed CR or M^n+^.

### 2.5. Linearity of UV-Vis Method

**Figure 1:**
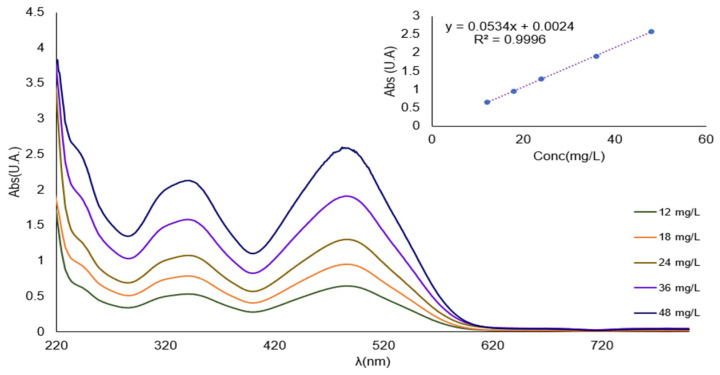
The UV–Vis spectra of CR were recorded over the 200–800 nm range to assess the linearity of the spectrophotometric method.

To ensure accurate quantification of CR after functionalization of MS, a UV-Vis linearity was studied. For this, the linearity of the UV–Vis method was evaluated in the concentration range 12–48 mg/L CR (Figure 1) at λ = 490 nm. The calibration plot of absorbance (A) at nm vs. concentration (C) yielded the regression line: A = 0.0534C + 0.0024 with a correlation coefficient of R^2^ = 0.9996. The excellent linearity confirms the validity of Beer’s law across the studied range and supports the reliability of the spectrometric determinations.

### 2.6. Procedure Used to Functionalize Maize Stalk with CR

The functionalization procedure was performed by adding 0.02 L of a Congo red (CR) solution (initial concentration: 120 mg L^−1^) over 0.05 g of MS in Erlenmeyer flasks. The pH of the working solutions was adjusted to the selected values (2.0, 4.0, 6.0, and 10.0). The mixtures were stirred at 175 rpm for 60 min at T = 25 ± 2 °C [26,27]. At the end of contact time, the mixture was filtered, and the CR concentration in the filtrate was determined spectrophotometrically. Based on these measurements, the adsorption capacity Q_e_ (mg g^−1^) was calculated according to Equation (1), and the CR removal efficiency R (%) according to Equation (2) [28,29,30,31,32]:

(1) $$Q_{e}=\frac{(C_{i}-C_{e})\cdot V}{m}$$

(2) $$R(\%)=\frac{C_{i}-C_{e}}{C_{i}}\times 100$$

where *C*_i_ and *C*_e_ (mg/L) are the initial and equilibrium concentrations of CR, V (L) is the solution volume, and m (g) is the dry mass of MS. The buffer solutions employed for pH adjustment were selected to ensure stable and well-defined working conditions: phosphate buffer for pH 2.0, acetate buffer for pH 4.0 and 6.0, phosphate buffer for pH 8.0, and carbonate buffer for pH 10.0.

For functionalization, a concentration of 120 mg/L of CR was chosen to ensure sufficient driving force for CR adsorption, ensuring efficient loading of the surface with functional groups without saturating the MS matrix. This concentration falls within the linear spectrophotometric method range, allowing for precise quantification of the retention of CR while ensuring that pH acts as the sole controlled variable during functionalization.

### 2.7. Procedure for Evaluating the Stability of MS–CR Material

The stability of the MS–CR material was evaluated under acidic and strongly alkaline conditions. For this purpose, 0.50 g of MS–CR loaded with 48 mg CR/g MS was weighed into Erlenmeyer flasks. Subsequently, 0.03 L of 2 M HCl and 2 M NaOH was added to each sample to assess the desorption of CR. The mixtures were stirred at 175 rpm for 30 min at T = 25 ± 2 °C to ensure complete interaction between the functionalized biomass and the acidic or alkaline medium. After stirring, the mixture was allowed to stand for 5 min, followed by vacuum filtration. The filtrates were analyzed by the UV–Vis method in the 200–800 nm range to quantify CR concentration released into solution. The stability of the MS–CR material was assessed by comparing the absorbance values of the filtrates with the calibration curve and calculating the quantity of dye desorbed under each condition.

### 2.8. Experimental Procedure for the Adsorption of Fe^3+^, Cr^3+^ and Mn^2+^ as a Function of Solution pH

The batch experiments were carried out to evaluate the influence of solution pH on the retention of Fe^3+^, Cr^3+^ and Mn^2+^ ions onto the MS–CR. For each experiment, 0.05 g of MS–CR (loaded with 48 mg CR/g MS) was added to 0.02 L of metal ion solution with an initial concentration of 2 mg/L. The pH of the working solutions was adjusted to the selected values (pH 2.0, 4.0, 6.0, 8.0 and 10.0) using appropriate buffer systems to ensure stable and well-defined conditions. The mixtures were stirred at 175 rpm for 60 min at T = 25 ± 2 °C to allow adsorption equilibrium [27,33]. After each contact time, the suspensions were filtered, and the residual concentrations of Fe^3+^, Cr^3+^, and Mn^2+^ in the filtrates were determined by atomic absorption spectrometry (AAS) in the 1–5 mg/L range. The removal efficiency, R (%), was calculated according to Equation (2). The effect of pH on metal ion retention was assessed by comparing the adsorption parameters obtained for each pH value.

### 2.9. Procedure for Studying the Influence of Contact Time on Fe^3+^, Cr^3+^, and Mn^2+^ Adsorption on MS–CR Mass

The effect of contact time on the adsorption of Fe^3+^, Cr^3+^, and Mn^2+^ onto MS–CR was investigated in batch mode at pH 10. For each mixture, aqueous solutions with C_i_ = 5 mg/L M^n+^ were prepared using an acetate buffer to maintain a constant pH = 10. In each experiment, 0.05 g of MS–CR was added to 0.02 L of metal solution in Erlenmeyer flasks. The mixtures were stirred at 175 rpm (T = 25 ± 2 °C) and, at different contact times of 15, 30, 45, 60, 75, and 90 min. At each selected time, the mixtures were filtered, and the concentrations at time t, of Fe^3+^, Cr^3+^, and Mn^2+^ in the filtrates were determined by atomic absorption spectrometry (AAS). The removal efficiency, R (%), was calculated according to Equation (2), whereas the adsorption capacity at time t, Q_t_ (mg/g), was determined according to Equation (3) [34,35].

(3) $$Q_{t}=\frac{(C_{i}-C_{t})\cdot V}{m}$$

where C_i_ and C_t_ (mg/L) represent the concentrations of Fe^3+^, Cr^3+^, and Mn^2+^ ions at the beginning of the experiment (i) and at time t, respectively; m (g) is the mass of MS–CR used, and V (L) is the volume of the metal ion solution.

### 2.10. Procedure for Studying the Influence of Initial Concentration on the Adsorption of Fe^3+^, Cr^3+^, and Mn^2+^ onto MS–CR

Batch adsorption experiments were conducted to evaluate the influence of the initial concentration of Fe^3+^, Cr^3+^, and Mn^2+^ ions on the adsorption capacity of the MS–CR material. All experiments were performed at pH 10, which was maintained using an acetate buffer. The metal solutions were prepared at initial concentrations of 0.5, 1, 1.5, 2, 4, 5, 6, 7, 8, 9, and 10 mg/L, and 0.02 L of each solution was placed in individual Erlenmeyer flasks. Subsequently, 0.05 g of MS–CR was added to each flask, maintaining the same adsorbent dose for all experiments. The mixtures were agitated for 60 min at 175 rpm, at T = 25 ± 2 °C, to allow the adsorption equilibrium to be reached. After agitation, the solid phase was separated by filtration.

The C_e_ of Fe^3+^, Cr^3+^, and Mn^2+^ in the filtrates was determined by the AAS method. The Q_e_ and R (%) were calculated based on Equations (1) and (2).

### 2.11. Procedure for Desorption of Metal Ions from MS–CR Mass

The desorption behavior of Fe^3+^, Cr^3+^ and Mn^2+^ from MS–CR was evaluated using acidic solutions of different strengths. MS–CR samples previously loaded with 1.98 mg/g Fe^3+^, 1.64 mg/g Cr^3+^ and 1.46 mg/g Mn^2+^ were used for the desorption experiments. For each experiment, ≈0.05 g of material metal loaded (MS–CR-M^n+^) was contacted with 30 mL of HCl solutions of 0.25, 0.5 and 1 M in Erlenmeyer flasks. The mixtures were stirred in batch mode at 175 rpm for 30 min at T = 25 ± 2 °C [26,36]. After stirring, the mixtures were allowed to stand for 5 min and then filtered. The filtrates were analyzed by AAS to determine the concentrations of Fe^3+^, Cr^3+^ and Mn^2+^ released into solution. The desorption efficiency (D%) of Fe^3+^, Cr^3+^ and Mn^2+^ from MS–CR was calculated according to Equation (4).

(4) $$D\;(\%)=\frac{M^{n+}des}{M^{n+}ads}\times 100$$

where M^n+^ des is the mass of metal desorbed in solution (mg) and M^n+^ ads is the mass of metal initially adsorbed on MS–CR (mg).

## 3. Results

### 3.1. Functionalization of MS with Complexing Agent CR in Function of pH Medium

**Figure 2:**
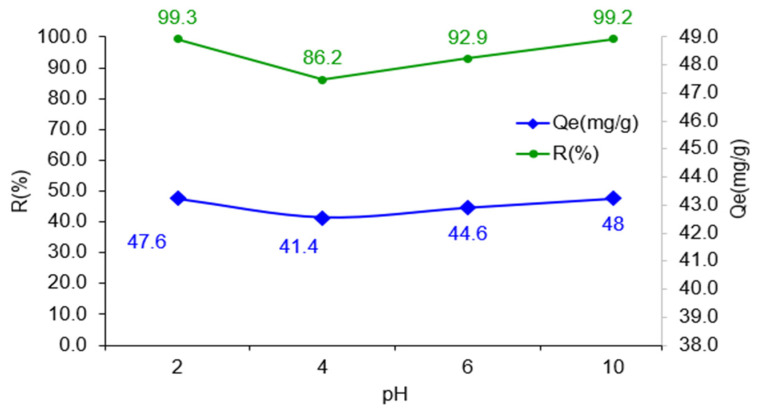
Influence of solution pH on the process functionalization of MS with CR. All measurements represent the average of two parallel experiments, each showing a standard deviation lower than 3%.

The adsorption capacity of CR on MS ranged from 41.4 to 48.0 mg/g over the pH range of 2–10, indicating a moderate effect of pH on the process, but sufficient to highlight the role of cellulose functional groups in the retention mechanism (Figure 2).

At pH 2, the Q_e_ value of 47.6 mg/g may be due to the protonation of hydroxyl groups in the cellulose structure, which generates a positively charged surface, favorable for electrostatic attraction toward the anionic dye. This electrostatic interaction is dominant in an acidic environment and leads to efficient retention.

At pH 4, the slow decrease of Q_e_ to 41.4 mg/g reflects a reduction in the degree of protonation and the emergence of a region where electrostatic interactions are diminished, and H^+^ ions compete with dye molecules for access to active sites.

At pH 6, the slight increase of Q_e_ to 44.6 mg/g indicated that, as electrostatic interactions diminish, cellulose-specific interactions become dominant, particularly the hydrogen bonds between the –OH groups and the azo and sulfonate functionalities of the CR complexing agent. These interactions are supported by the cellulose structure, which allows for the stabilization of the adsorbed molecules.

At pH 10, Q_e_ reached a maximum value of 48.0 mg/g, indicating that, in an alkaline environment, although cellulose is deprotonated and negatively charged, adsorption remains effective due to non-electrostatic interactions: the reorganization of the hydrogen bond network, increased accessibility of amorphous regions, and hydrophobic interactions with the dye’s aromatic structures. The contribution of lignin in the plant matrix can enhance retention through π–π interactions [33,37,38].

Thus, the adsorption profile demonstrates that CR retention on MS is controlled by a mixed mechanism, in which electrostatic interaction dominates at acidic pH, while at neutral–alkaline pH, interactions specific to cellulose and the lignocellulosic components in the MS structure are involved. The stability of the Q_e_ over the entire pH range of 2–10 confirms the material’s robustness and its potential for applications in environments with variable pH. After CR adsorption on the MS matrix, it is essential to evaluate the stability of the immobilized CR, as the material will subsequently be exposed to metal ion adsorption conditions in which wastewater streams may exhibit either acidic or alkaline pH values. In this context, the concentration of CR remaining in the MS mass after treatment with 2 M NaOH/HCl and this was below the detection limit of the analytical method.

The desorption experiments confirmed that CR was strongly retained on the MS matrix, indicating that it is fixed through strong π–π interactions with the aromatic domains of lignin, extensive hydrogen bonding networks, and partial physical entrapment within the lignocellulosic pores, which cannot be disrupted even under extreme acidic or alkaline conditions [33]. Taking into account that the MS-CR material has demonstrated high stability under both acidic and alkaline conditions, that it successfully passed the stability test, and that the highest CR loading was achieved at pH 10, the functionalization step was carried out at this pH to produce a sufficient amount of material for subsequent studies on metal retention.

### 3.2. pH Influence on Metal Adsorption by Complexing Material

**Figure 3:**
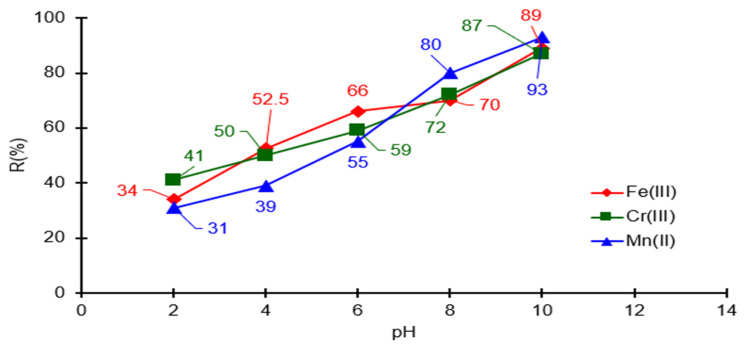
Influence of pH solution regarding adsorption of M^n+^ onto MS-CR. All measurements represent the average of two parallel experiments, each showing a standard deviation lower than 3%.

The solution of pH indeed has a pronounced influence on the adsorption of Fe^3+^, Cr^3+^ and Mn^2+^ ions onto MS-CR, as reflected by the systematic increase in removal efficiencies from pH 2 to 10 (Figure 3) [25,39,40].

Under strongly acidic conditions (pH 2, phosphate buffer), the MS-CR surface is extensively protonated, which limits the availability of active sites and results in low retention ≈ 31–41%. Among the three ions, Cr^3+^ exhibits slightly higher uptake, consistent with its strong affinity for oxygen-donor ligands even in acidic media.

At pH 4 and 6 (acetate buffers), the R (%) increases substantially between 39 and 66%. In this pH range, partial deprotonation of surface functional groups enhances electrostatic attraction and coordination interactions between the M^n+^ and the MS-CR matrix. Fe^3+^ shows the highest adsorption, followed by Cr^3+^ and Mn^2+^, reflecting its higher charge density and stronger interaction with the functionalized lignocellulosic surface. A further increase to pH 8 (phosphate buffer) and pH 10 (carbonate buffer) leads to high removal efficiencies for all ions (70–93%). At these pH values, the MS-CR surface becomes strongly negatively charged, which markedly favors the interaction with both trivalent and divalent ions. Mn^2+^ becomes the most efficiently retained species at basic pH (80–93%) at C_i_ = 2 mg/L, indicating that electrostatic attraction dominates the adsorption mechanism under these conditions and that the MS-CR surface exhibits high affinity for divalent ions when was fully deprotonated. Overall, the results demonstrate a clear pH-dependent enhancement of adsorption capacity, governed by the progressive deprotonation of MS-CR functional groups and the corresponding increase in electrostatic and coordination interactions. The distinct trends observed for Fe^3+^, Cr^3+^ and Mn^2+^ reflect differences in ionic charge, polarizability, and ligand affinity, confirming the strong pH sensitivity of the MS-CR adsorption system.

### 3.3. Effect of Contact Time of M^n+^ Adsorption on MS-CR Matrices in Batch Conditions

**Figure 4:**
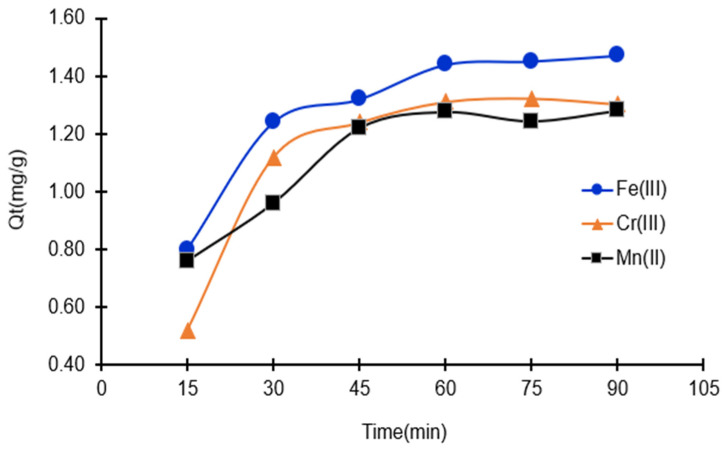
Effect of contact time on metal ion adsorption. All measurements represent the average of two parallel experiments, each showing a standard deviation lower than 3%.

The contact time between the liquid and solid phases is a key parameter that governs the adsorption process, as it dictates the rate at which metal ions migrate toward the active sites of the MS–CR material and form stable complexes [41,42,43,44,45,46]. To evaluate the kinetic behavior of Fe^3+^, Cr^3+^ and Mn^2+^, batch experiments were performed at pH 10 (acetate buffer), using C_i_ = 5 mg/L, 0.05 g MS–CR, and 0.02 L solution. The contact time was varied from 15.45, 60.75 to 90 min, using a stirring speed of 175 rpm (T = 25 ± 2 °C). The adsorption data for the retention of Fe^3+^, Cr^3+^ and Mn^2+^ as a function of contact time, are presented in Figure 4, indicated a rapid increase in adsorption at short contact times, followed by the attainment of characteristic equilibrium plateaus.

For Fe^3+^, the Q_t_ (mg/g) increased rapidly during the initial stage, from 0.80 mg/g at 15 min to 1.24 mg/g at 30 min, reaching 1.44 mg/g at 60 min. The corresponding removal efficiencies increased from 40% to 72%. After 60 min, the adsorption rate slowed considerably, with Qt reaching 1.47 mg/g at 90 min.

In the case of Cr^3+^, the adsorption capacity increased from Q_t_ = 0.52 mg/g at 15 min to 1.12 mg/g at 30 min, reaching 1.31 mg/g at 60 min (R = 65.6%). Beyond this point, only slight variations were observed, with Q_t_ values of 1.32 mg/g at 75 min and 1.30 mg/g at 90 min, confirming that equilibrium was reached in the first 60 min.

For Mn^2+^, the adsorption also followed a two-stage kinetic profile. Q_t_ increased from 0.76 mg/g at 15 min to 0.96 mg/g at 30 min, reaching 1.28 mg/g at 60 min (R = 61.8%). After 60 min, Q_t_ remains constant, obtaining 1.28 mg/g at 90 min.

Considering the kinetic profiles obtained for Fe^3+^, Cr^3+^, and Mn^2+^, which show a pronounced increase in Q_t_ during the first stage of the process, followed by only marginal variations between 60 and 90 min, the contact time of 60 min was identified as the optimal equilibrium time for subsequent adsorption experiments.

### 3.4. Adsorption of Metal Ions onto MS-CR Mass in Function of Initial Concentrations

The adsorption of Fe^3+^ on the MS-CR material shows its dependence on the solution pH. Thus, the results obtained at pH 10 (acetate buffer) show significantly higher performance compared to the acidic medium, as was presented in the previous Section 3.2.

**Figure 5:**
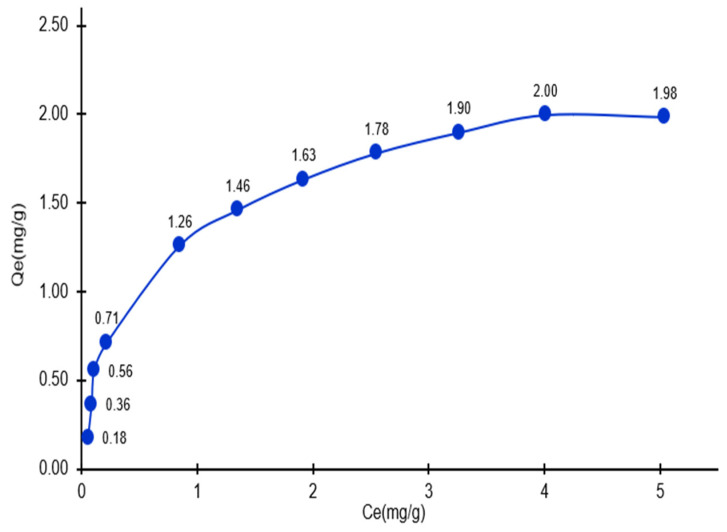
Experimental isotherm for Fe^3+^ adsorption on MS-CR. All measurements represent the average of two parallel experiments, each showing a standard deviation lower than 3%.

For a concentration range of 0.5–10 mg/L, a Q_e_ ranging from 0.18 to 2.00 mg/g and R (%) between 89 and 50% (Figure 5) were obtained.

At low concentrations (C_i_ ≤ 1.5 mg/L), MS-CR exhibits R (%) between 89 and 93% and Q_e_ increasing almost linearly from 0.18 to 0.56 mg/g, indicating the availability of a large number of accessible active sites. This operating range highlights strong Fe^3+^–ligand interactions, dominated by the electrostatic attraction between the strongly deprotonated surface (–SO_3_^−^, –O^−^) and Fe^3+^.

As the initial concentration increases (C_i_ = 2–10 mg/L), Q_e_ continues to increase up to 2.00 mg/g, reaching a plateau at high metal loading values, suggesting that the surface is approaching saturation. In this region, the removal efficiency gradually decreases from 89% to 50%, a behavior typical of adsorbents with a finite number of active sites.

However, the continued increase in Q*_e_* demonstrates that complexation and coordination mechanisms remain active even at high concentrations, exceeding the purely electrostatic contribution, functionalization. The introduced sulfonic groups (–SO_3_H to –SO_3_^−^ at alkaline pH) increase the negative charge density, promoting interaction with Fe^3+^ ions, which are known for their high affinity for anionic surfaces [40,47,48]. Furthermore, the aromatic structure of the dye may facilitate chelation or π-assisted coordination processes, contributing to the high adsorption capacity values (up to 2.00 mg/g), which are superior to those of non-functionalized lignocellulosic materials reported in the literature [49,50].

Overall, the results demonstrate that MS-CR is a highly efficient adsorbent for the removal of Fe^3+^ under alkaline conditions, where both the surface chemistry and the ionic form of Fe^3+^ develop strong interactions and complex formation. The clear contrast between the performance at low pH and at pH 10 emphasizes the importance of pH optimization in practical applications and confirms the advantage of functionalization in increasing the affinity of biomass for trivalent metal ions.

**Figure 6:**
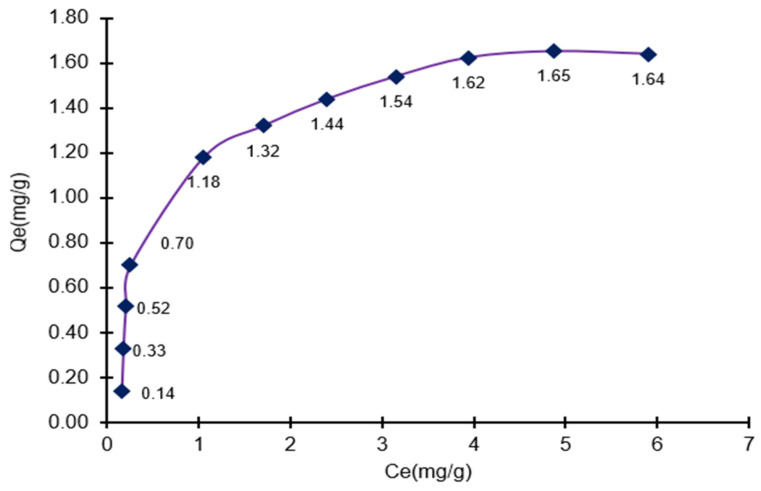
Experimental isotherm for Cr^3+^ adsorption on MS-CR. All measurements represent the average of two parallel experiments, each showing a standard deviation lower than 3%.

For Cr^3+^ adsorption on MS-CR mass, the Q_e_ was varied between 0.33 and 1.64 mg/g for C_i_ = 1–10 mg/L, while the removal efficiency gradually decreases from 83% to 41% as the initial concentration increases (Figure 6).

At low C_i_ = 1–2 mg/L, MS-CR exhibits a removal efficiency between 83 and 87% and Q_e_ values were increased from 0.33 to 0.70 mg/g. Furthermore, the azo groups (–N=N–) in the CR structure contribute to the stabilization of the complexes through electronic delocalization effects and can act as electron transfer bridges, facilitating the coordination of Cr^3+^ in the vicinity of donor centers (–SO_3_^−^, –NH–, –O^−^). This structural synergy explains the high efficiencies observed at low concentrations.

As the initial concentration increases (C_i_ = 4–10 mg/L), Q_e_ continues to increase from 1.18 to 1.64 mg/g, approaching a plateau at high metal loadings, suggesting that the surface is nearing saturation. In this region, the removal efficiency decreases progressively from 74% to 41%, a behavior typical of adsorbents with a finite number of active sites.

However, the continued increase in Q_e_ demonstrates that complexation and ligand exchange mechanisms remain active even at high concentrations, exceeding the purely electrostatic contribution. The superior performance at pH 10 highlights the essential role of CR functionalization. The sulfonic groups from the CR structure (–SO_3_H → –SO_3_^−^) significantly increase the negative charge density in an alkaline medium, facilitating interaction with the positive Cr^3+^ species. The azo groups, due to their conjugated nature and ability to stabilize charges through resonance, can contribute to the formation of more stable complexes, either through inductive effects or by facilitating chelation in the vicinity of the donor groups.

In addition, the aromatic structure and nitrogen-containing groups of the CR can promote chelation processes, contributing to the stability of the formed complexes [40,51,52,53,54,55,56]. These structural characteristics explain the adsorption capacity values (up to 1.64 mg/g) and the superior performance compared to non-functionalized lignocellulosic materials reported in the literature. Overall, the results demonstrate that MS-CR is an effective adsorbent for Cr^3+^ in the range of 1–10 mg/L, with optimal performance under alkaline conditions. The combination of surface deprotonation, enhanced electrostatic attraction, the contribution of azo groups to complex stabilization, and strong complexation mechanisms drives the improved adsorption behavior.

The contrast between low and high concentrations highlights the importance of surface charge modulation and metal speciation in determining adsorption efficiency, confirming the high affinity of the functionalized material in the presence of Cr^3+^ ions.

**Figure 7:**
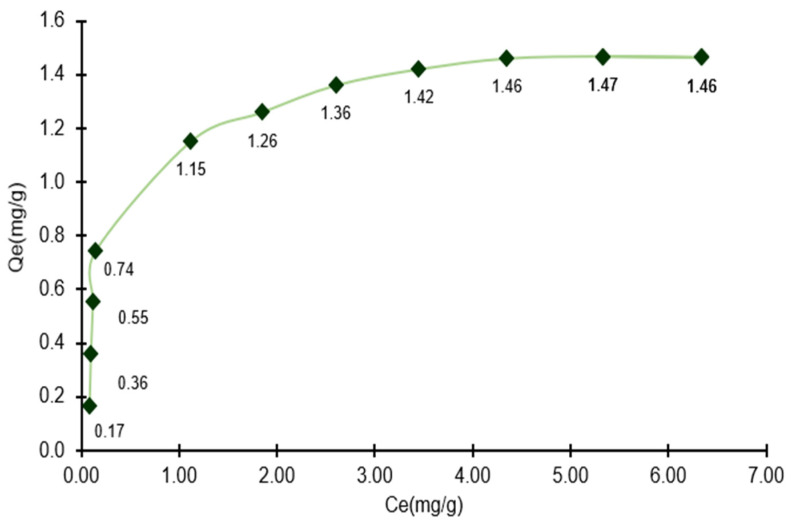
Experimental isotherm for Mn^2+^ adsorption on MS-CR. All measurements represent the average of two parallel experiments, each showing a standard deviation lower than 3%.

The adsorption of Mn^2+^ onto MS-CR showed Q**_e_** values that ranged from 0.36 to 1.46 mg/g, with corresponding removal efficiencies decreasing from 91% to 37% as the initial Mn^2+^ concentration increased from 1 to 10 mg/L (Figure 7).

At low concentrations (C_i_ = 1–2 mg/L), MS-CR exhibits high efficiency, with R (%) between 91 and 93% and Q_e_ values increasing from 0.36 to 0.74 mg/g, indicating the availability of a large number of accessible active sites.

In the alkaline medium, the MS-CR surface is strongly deprotonated, and the sulfonate (–SO_3_^−^), phenolate (–O^−^), and carboxylate (–COO^−^) groups contribute to the electrostatic attraction toward the M^n+^ ion [26,27,57].

Azo groups (–N=N–), located within a conjugated aromatic system, play an additional role by increasing the local electron density and facilitating Mn^2+^ coordination [57,58,59].

Although Mn^2+^ forms weaker complexes than Fe^3+^ or Cr^3+^, the presence of these groups contributes to the stabilization of metal–ligand interactions in the vicinity of donor centers. As the initial concentration increases (C_i_ = 4–10 mg/L), Q_e_ continues to increase from 1.15 to 1.46 mg/g, reaching a saturation plateau. In this region, the removal efficiency decreases progressively from 72% to 37%. However, the continued increase in Q_e_ demonstrates that ligand-assisted coordination mechanisms remain active even at high concentrations, surpassing the purely electrostatic contribution.

The superior performance at pH 10 highlights the essential role of CR functionalization.

CR introduces: (i) sulfonic groups (–SO_3_^−^), which provide strong anionic sites for M^n+^ binding; (ii) azo groups (–N=N–), which, through electronic delocalization and coordination potential, contribute to the stabilization of Mn^2+^ complexes; (iii) aromatic nuclei, which can facilitate additional interactions through resonance effects.

### 3.5. Desorption of M^n+^ from Saturated MS-CR Mass

**Figure 8:**
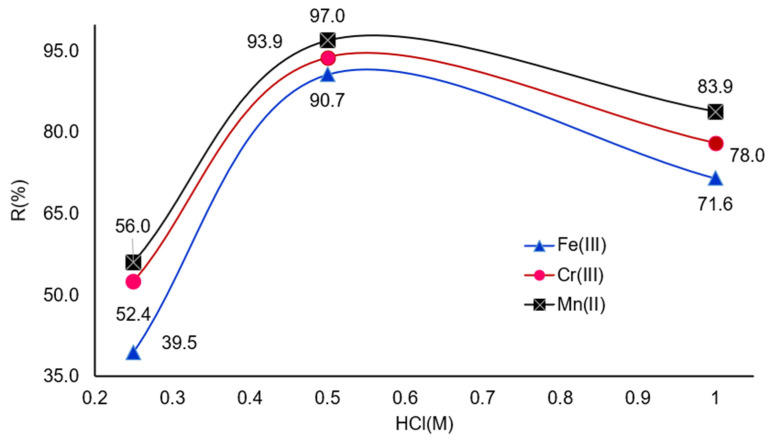
Desorption of M^n+^ from MS-CR mass. All measurements represent the average of two parallel experiments, each showing a standard deviation lower than 3%.

The desorption of Fe^3+^, Cr^3+^, and Mn^2+^ from MS-CR was studied using HCl solution as the desorption agent [60,61,62,63,64]. The experimental results indicated a nonlinear dependence of desorption when different HCl concentrations were used (Figure 8).

At 0.25 M HCl, the desorption of M^n+^ was 39.5% for Fe^3+^, 52.4% for Cr^3+^, and 56% for Mn^2+^. This behavior can be attributed to insufficient protonation of the active functional groups on the MS-CR surface (–SO_3_^−^, –O^−^, –NH–), which allowed a significant fraction of the binding sites to remain negatively charged and capable of retaining M^n+^. Furthermore, competition between H^+^ and M^n+^ was relatively weak, and the CR-M^n+^ complexes remained relatively stable under these conditions. When the eluent concentration was increased to 0.5 M HCl, the desorption percentages reached 90.7% for Fe^3+^, 93.9% for Cr^3+^, and 97.0% for Mn^2+^. At this concentration, HCl provides sufficient acidic strength to extensively protonate the anionic functional groups, destabilize the metal–ligand complexes, and promote effective competition of H^+^ ions for the adsorption sites.

At 1 M HCl, the M^n+^ desorption was 71.6% for Fe^3+^, 78.0% for Cr^3+^, and 83.9% for Mn^2+^, despite the higher acidity. This behavior can be attributed to the negative effects of excessive protonation: over protonation can cause partial compaction of the lignocellulosic matrix, reducing the diffusional accessibility of metal ions.

In addition, the very high ionic strength compresses the electric double layer and can generate a dense layer of H^+^ and Cl^−^ ions at the surface (“ionic agglomeration”), which prevents the release of M^n+^. Under these conditions, the mobility of metal species decreases, resulting in lower desorption efficiency compared to the 0.5 M HCl concentration.

Overall, the desorption efficiency follows the order: Mn^2+^ > Cr^3+^ > Fe^3+^, reflecting the different stabilities of the MS-CR-M^n+^ complexes and the mobility of M^n+^ from the MS matrix. To conclude, 0.5 M HCl offers the most favorable balance between acidity, structural preservation, and metal-ion mobility, accounting for the maximum desorption observed.

### 3.6. FTIR-ATR Analysis

#### 3.6.1. FTIR Spectra of MS-CR

**Figure 9:**
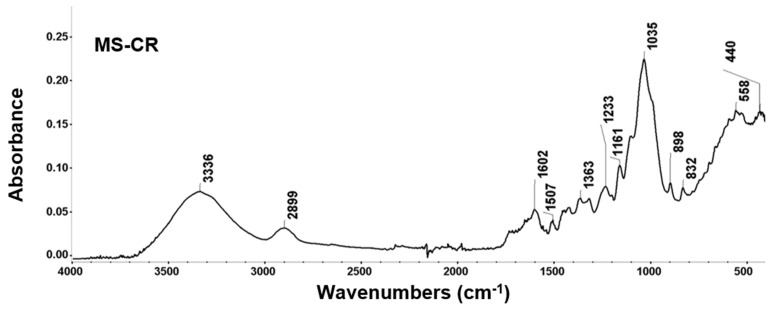
FTIR spectrum for MS-CR.

FTIR spectra of MS matrix show the characteristic of cellulose, hemicellulose, and lignin bands, along with new or intensified aromatic and amine-related peaks (1500–800 cm^−1^), consistent with CR adsorption (Figure 9) [65,66,67].

Table 1 shows the assignment of functional groups to the peaks identified in the FTIR spectrum for the adsorption of CR on MS mass [66,68].

The broad O–H band (around 3335 cm^−1^) and C–H stretch (2899 cm^−1^) show the presence of polysaccharide material (cellulose/hemicellulose) [65,66,67].

The bands between 1600 and 1500 cm^−1^ reflect aromatic structures from lignin and CR, which both contain aromatic rings [69,70].

The 1034–1106 cm^−1^ area is rich in C–O and C–O–C vibrations typical of polysaccharides.

The 898 cm^−1^ band (β-linkage) is diagnostic of cellulose [66].

The additional aromatic and C–N bending peaks (around 1333–812 cm^−1^) show CR adsorption on the MS material [65,68].

Presentation of the main bands:

The broad absorption band around ~3350 cm^−1^ can be assigned to O–H stretching vibration from cellulose/hemicellulose and adsorbed water, and it can also indicate possible hydrogen bond formation between the MS and CR [65,68].

The asymmetric and symmetric stretching vibration of C–H bonds in methylene (-CH_2_) groups in the cellulose chains or other sp^3^ carbons can be observed at ~2920–2850 cm^−1^. In fingerprint region of ~1600–1640 cm^−1^ (e.g., ~1601–1609 cm^−1^), C=C vibrations or vibrations associated with conjugated bonds may reflect aromatic groups from lignin or benzene groups in the CR structure [69,70,71].

The peak from ~1510 cm^−1^ is characteristic of the aromatic skeleton and is typical for lignin (indicating the presence of aromatic components) [66].

The C–O–C or C–O (ester/ether) stretching vibrations associated with hemicellulose, lignin, or ether linkages appears at ~1230–1250 cm^−1^ and may indicate bonds between the functional groups of the CR and the polymeric groups of the MS [66,68]. At the same time, the strong peak from region 1030–1050 cm^−1^ can be assigned to C–O (in primary alcohol) and C–O–C stretching vibration from cellulose, confirming the presence of the polysaccharide structure [66].

Below 900 cm^−1^ down to 600 cm^−1^ are assigned to deformation vibrations, possible out-of-plane bands for aromatic substituents or signals of sulfonate/azo groups in the complexing agents (CR contains sulfonate and azo groups) [65,68].

#### 3.6.2. Observations Related to the Interaction Between MS and Complexing Agents

The presence of a broad O–H band and possibly changes in the position/intensity of the C–O/C–O–C bands suggest hydrogen bond formation between the hydroxyl groups of cellulose/hemicellulose and the azo/sulfonate/aromatic groups of CR structure [65,68,70].

Aromatic bands (~1600–1500 cm^−1^) may reflect both stem lignin and the aromatic signature of the CR; a possible overlap of the bands indicates adsorption or physicochemical association.

The FTIR data suggest that CR has interacted with MS components. Major peaks indicate the presence of hydroxyl groups, aliphatic C–H bonds, azo/aromatic groups (lignin), and cellulose/hemicellulose C–O bonds. Bands are also observed that may reflect interactions between the lignocellulosic material matrix (MS) and the adsorbed CR. The spectrum indicates typical components of lignocellulosic material (O–H, C–H, C–O/C–O–C, aromatic vibrations) and signals compatible with the presence of CR (aromatic, possibly sulfonate/azo).

**Figure 10:**
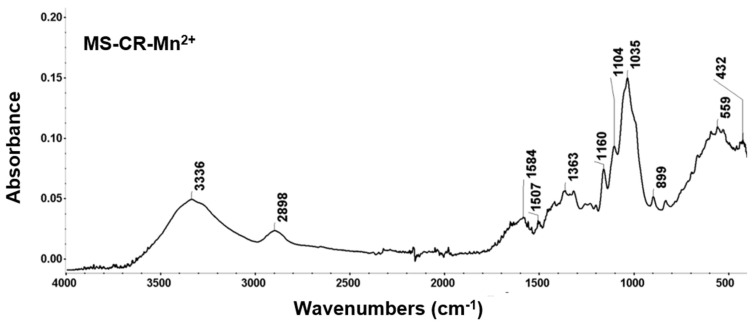
FTIR spectrum for MS-CR-Mn^2+^.

The intensity changes and overlaps suggest physicochemical interactions (hydrogen bonds, electrostatic/aromatic interactions) between the MS and the CR. FTIR spectroscopy identifies chemical functional groups based on molecular vibrations at specific wavelengths, expressed in cm^−1^, of the main absorption bands visible in Figure 10 [53,54].

Although all three metal ions (Fe^3+^, Cr^3+^, and Mn^2+^) were retained by the MS-CR material and the resulting solid phases (CR-MS-M^n+^) were analyzed by FTIR-ATR, only Mn^2+^ induced detectable structural modifications in the FTIR spectra of the MS-CR–Mn^2+^ system.

#### 3.6.3. Single Hydrogen Bond Area (4000–2500 cm^−1^)

3335.96 cm^−1^: Intense broad band. Specific to the stretching vibration of hydroxyl groups (-OH) in cellulose, hemicellulose and lignin (components of MS). May also indicate (-NH) bonds in the structure of CR or residual water molecules [66,68];

2898.09 cm^−1^: Band characteristic of the C-H stretching vibration of aliphatic groups (-CH_2_ and -CH_3_), present in polysaccharides of plant biomass [66].

Double bond area (1700–1500 cm^−1^):

1594.16 cm^−1^: Stretching vibration of aromatic bonds (C=C) in the lignin structure. This band is often also accentuated by vibrations of aromatic nuclei or azo groups (-N=N-) in the CR molecule [68,69]; 1506.66 cm^−1^: Secondary vibration of the aromatic ring in lignin or in the structure of the aromatic CR.

#### 3.6.4. Fingerprint Area (1500–900 cm^−1^)

1363.13 cm^−1^: C-H deformation vibration in cellulose and hemicellulose;

1160.28 cm^−1^: Asymmetric stretching vibration of the C-O-C ester or ether bond, typical of the carbohydrate structure of biomass;

1104.46 cm^−1^: Vibration associated with deformations of the glucose ring in cellulose;

1034.83 cm^−1^: The most intense peak in the spectrum. It is the predominant band of cellulose, corresponding to the C-O and C-C stretching vibration in the polysaccharide backbone. The presence of metals and CR may cause slight shifts or intensity changes of this peak through coordination/adsorption [72,73,74].

#### 3.6.5. Low Wavenumber Area (<900 cm^−1^)

898.65 cm^−1^: Band associated with β-glycosidic bonds between glucose units in cellulose;

559.37 cm^−1^: The area below 600 cm^−1^ often corresponds to organic lattice deformation vibrations or metal–oxygen (Mn^2+^-O) bond vibrations, suggesting chemical interaction (complexation or adsorption) of manganese ions (Mn^2+^) on the biomass surface.

**Figure 11:**
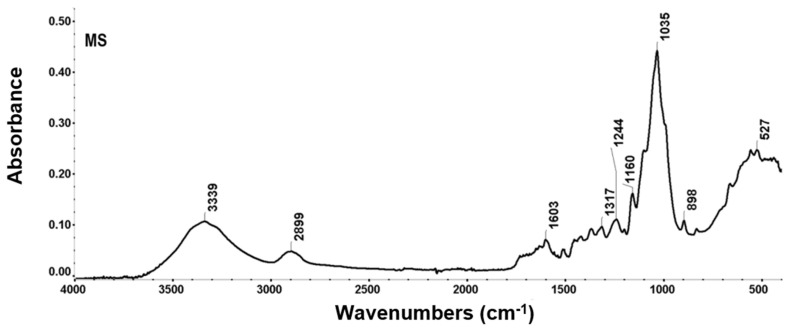
FTIR spectrum for MS.

To clarify the adsorption and chemical interaction process, we compared the MS spectrum (Figure 11) with the MS-CR-Mn^2+^ spectrum (Figure 10).

The disappearance of the carboxyl band at 1243 cm^−1^ and the emergence of the metal–oxygen vibration at 559 cm^−1^ confirm the coordination of Mn^2+^ to deprotonated carboxyl and phenolic groups. The analysis of the intensity changes and peak shifts highlights the binding mechanisms of CR and Mn^2+^ on biomass [70,72,73].

#### 3.6.6. Area of Hydroxyl (-OH) and Amine (-NH) Groups

Shift to lower wavenumbers (from 3338.91 cm^−1^ to 3335.96 cm^−1^), displacement of the vibration band from its initial position (Dn) was −3 cm^−1^ and a slight flattening of the band.

This shift indicates the involvement of aliphatic hydroxyl groups in maize stalk cellulose in the formation of new hydrogen bonds or electrostatic interactions with the amine (-NH_2_) and sulfonic (SO_3_^−^) groups of CR, as well as coordination with the metal ion Mn^2+^ [65,68,72,73].

#### 3.6.7. Area of Double Bonds and Aromatic Rings (C=O, C=C, N=N)

Major structural shift in the region 1600–1500 cm^−1^; 1602.78 cm^−1^ (control sample): weak/wet vibration, can be attributed to adsorbed water and lignin. A clear peak appears at 1594.16 cm^−1^ and a new peak at 1506.66 cm^−1^. The appearance and shift of these bands confirm the retention of CR dye on the biomass surface.

The band at 1594 cm^−1^ corresponds to vibrations of the aromatic skeleton (naphthyl and phenyl rings in the dye) and azo bonds (-N=N-). The shape change suggests π-π interactions between the aromatic rings of corn lignin and those of the dye [70,75].

#### 3.6.8. Area of Aliphatic Groups and Carboxylic Acids

MS spectrum: shows a distinct peak at 1243.60 cm^−1^ (attributed to the stretching vibration of the acetyl groups in hemicellulose). MS-CR spectrum: this peak disappears or is completely masked, with a new peak appearing at 1363.13 cm^−1^ instead.

The disappearance or modification of the band at 1243 cm^−1^ suggests a local chemical modification or deprotonation of the carboxyl/ester groups, facilitating binding (chelation) sites for the studied metal ions (Mn^2+^) [72,73,74].

#### 3.6.9. Polysaccharide Backbone Area (C-O-C and C-O)

Peak 1160 cm^−1^ (ester/ether bonds): remains stable in position (1160.03–1160.28 cm^−1^), but decreases significantly in relative intensity.

The maximum peak 1034.89 cm^−1^ (C-O stretch from cellulose): Remains at an almost identical position (1034.83 cm^−1^), but the intensity ratio between this peak and the rest of the spectrum changes. The main crystalline structure of cellulose is not destroyed (so we do not have a severe chemical degradation of the biomass), but the decrease in relative intensities shows that the polysaccharide surface has been covered (screened) by a layer of CR and complexed metal ions [66,76].

#### 3.6.10. Low Footprint Area (Interaction with Metal)

Control spectrum: shows a peak at 526.65 cm^−1^. Treated spectrum: The peak shifts significantly to 559.37 cm^−1^ and becomes much more pronounced. This major shift in the low wavenumber region is direct evidence of heavy metal (Mn^2+^) biosorption. The bands in this region are associated with lattice deformation vibrations and the appearance of metal–oxygen (Mn-O) or metal–nitrogen (Mn-N) coordination bonds, through which the manganese ion is fixed to the modified biomass.

The FTIR spectrum of the MS-CR allows the analysis of the isolated effect of the CR from that of the heavy metal [65,70]. Comparing this spectrum with that of the simple MS and with that of the MS-CR-Mn^2+^, specific changes are observed that isolate the MS-CR interaction.

Area 3335.54 cm^−1^ (-OH/NH): The band shifts from 3338.91 cm^−1^ (control) to 3335.54 cm^−1^ and decreases in intensity (A = 0.08). This proves that the hydroxyl groups of cellulose are the main sites that form hydrogen bonds with the amine groups of CR [65,68].

The 1601.88 cm^−1^ region and the appearance of the band at 1506.99 cm^−1^: Unlike the control, where the signal at 1602 cm^−1^ was weak and diffuse, here a much better-defined profile appears and the secondary peak at 1506.99 cm^−1^ clearly develops. These bands can be attributed to the vibrations of the aromatic skeleton (C=C) and azo bonds (-N=N-) in the structure of CR, which shows the physicochemical adsorption of the CR.

Although the O–H stretching band exhibited only a minor shift (3338.91 → 3335.54 cm^−1^), close to the instrumental resolution limit (4 cm^−1^), more conclusive evidence of interaction is provided by the band at 1233.26 cm^−1^ (1243 cm^−1^ in native MS), assigned to carboxyl/ester groups of hemicellulose, which remains detectable in the MS–CR spectrum but disappears entirely in the MS–CR–Mn^2+^ spectrum, demonstrating selective involvement of carboxyl groups in metal coordination [72,73,74]. Additionally, a broad band centred at 558.50 cm^−1^, absent in native MS, emerges in the MS–CR spectrum and is attributed to deformation vibrations of the adsorbed CR molecule and its sulfonic (–SO_3_^−^) groups. Together, these resolution-independent changes provide robust support for the proposed MS–CR and MS–CR–Mn^2+^ interaction mechanisms.

#### 3.6.11. Characterization of Adsorption Mechanisms by FTIR Spectroscopy

To elucidate the intermolecular mechanisms that govern the simultaneous retention of organic pollutants and metal ions on MS matrix, comparative analysis of FTIR spectra was used [70,72,73]. Three experimental systems were investigated: native biomass (MS), binary system loaded MS-CR and MS-CR-Mn^2+^.

The modification of the positions and intensities of the absorption bands reflects the nature of the chemical interactions at the surface of the biosorbent (Table 2).

The hypsochromic shift of the broad band from 3338.91 cm^−1^ to 3335.54 cm^−1^ in the MS-CR system demonstrates the involvement of the hydroxyl groups of cellulose in the formation of hydrogen bonds with the functional groups of CR [65,68]. The CR loading is supported by the appearance and clear individualization of bands in the 1601–1506 cm^−1^ area, specific to the aromatic skeleton and azo bonds (-N=N-) [68,69]. A critical spectroscopic behavior occurs in the case of carboxylic groups (~1243 cm^−1^). While upon CR adsorption the band persists (suffering only a minor shift), in the presence of the heavy metal, it completely disappears from the spectrum. This phenomenon confirms the selective deprotonation of organic acids in hemicellulose and their exclusive involvement in the fixation of Mn^2+^ ions [72,73,74]. The argument is supported by the major band shift from 526.65 cm^−1^ to 559.37 cm^−1^ in the ternary system, attributed to the formation of new metal–oxygen (Mn-O) coordination bonds. The complete disappearance of the band at ~1243 cm^−1^ only in the manganese sample represents a strong argument to demonstrate the difference between physical (CR) and chemical/coordinative (metal) adsorption [72,73,74]. FTIR data indicate a combined biosorption/chemisorption process: CR attaches to the maize stalk through hydrogen bonds (-OH from cellulose) and hydrophobic/aromatic interactions (with lignin). Mn^2+^ ions are retained by chemical coordination (chelation), interacting with the active groups on the surface (the oxygen from -OH or carboxyl’s and the nitrogen from the azo groups of the adsorbed CR), a fact confirmed by the appearance of the band at 559 cm^−1^ [72,73,74]. To quantify the structural changes between the two spectra, we calculated the intensity (absorbance) ratios of some key bands. FTIR spectroscopy of biomass offers the possibility to measure crystallinity indices or the degree of chemical substitution on the surface. Thus, we correlated the approximate absorbance values (Y axis) for the main points of interest:

The ratio of active groups to the cellulose structure (A_3338_/A_1034_) measures the density of available hydroxyl groups relative to the stable cellulose matrix.

Control spectrum: simple MS at A_3338_ ≈ 0.11 and A_1034_ ≈ 0.44. Ratio control = 0.11/0.44 = 0.250

Treated spectrum (MS-CR-Mn^2+^) at A_3338_ ≈ 0.08 and A_1034_ ≈ 0.44. Ratio treated = 0.181.

The significant decrease in the ratio (from 0.250 to 0.181) mathematically demonstrates that a large portion of the free -OH groups were occupied or blocked following the binding of the dye and manganese ions, reducing the free hydrogen vibration [76].

The ratio of aromatic components/polysaccharides (A_1602_/A_1034_) shows the proportion of aromatic structures (lignin/dye) to carbohydrates in the biomass.

MS spectrum: at A_1602_ ≈ 0.05 and A_1034_ ≈ 0.44. Resulting ratio control = 0.05/0.44 = 0.113.

Treated spectrum (MS-CR-Mn^2+^) at A_1594_ ≈ 0.09 and A_1034_ ≈ 0.44. Resulting ratio treated = 0.09/0.44 = 0.204. The almost twofold increase in this ratio (from 0.113 to 0.204) quantitatively confirms the loading of the biomass with aromatic rings originating exclusively from the molecular structure of the CR retained by adsorption [65,70].

#### 3.6.12. Change in Relative Crystallinity Index (A_1365_/A_898_)

For the vegetable mass, the ratio between the band at ~1365 cm^−1^ (CH deformation) and that at ~898 cm^−1^ (β-glycosidic bond) indicates changes in the cellulose degradation or swelling treatment. In both spectra, the relative intensities of these two bands maintain their stable proportion with respect to the local baseline. The biosorption process of heavy metals and dyes took place strictly on the surface of the stalk (through chelation and physicochemical bonds), without destroying the internal crystalline structure of the maize fibers.

#### 3.6.13. Relative Hydrophobicity Index (RHI)

This index evaluates the balance between hydrophilic components (polar -OH groups) and hydrophobic ones (aliphatic -CH_2_ and -CH_3_ groups, or the aromatic nuclei of lignin and dye). It is frequently calculated by the ratio of the area or intensity of the aliphatic bands (~2898 cm^−1^) to that of the hydroxyl groups (~3335 cm^−1^); RHI = A _2898_/A_3335_.

Based on the absorbance values extracted from the spectra, we have:

MS: A_2898_ ≈ 0.028; A_3338_ ≈ 0.110; RHI = 0.254

MS-CR: A_2899_ ≈ 0.032; A_3335_ ≈ 0.082; RHI = 0.390

MS-CR-Mn^2+^: A_2898_ ≈ 0.029; A_3335_ ≈ 0.080; RHI = 0.362

The RHI increase from 0.254 (MS) to 0.390 (MS-CR) demonstrates that the fixation of CR on the MS surface blocks the hydrophilic -OH groups and exposes the hydrophobic aromatic nuclei of the CR. In the MS-CR-Mn^2+^ system, the value decreases slightly to 0.362 due to the presence of hydrated Mn^2+^ ions, which reintroduce a partial hydrophilic character into the surface network.

#### 3.6.14. Lateral Order Index (LOI)

This parameter is used to verify whether chemical treatment or adsorption has modified the deep crystalline structure of cellulose.

It is calculated as the ratio between the band sensitive to crystallinity (~1420–1430 cm^−1^ or the -CH_2_ deformation at ~1363 cm^−1^) and the amorphous band (~898 cm^−1^): LOI = A_1363_/A_898_.

MS: A_1365_ ≈ 0.061; A_898_ ≈ 0.062; LOI = 0.983;

MS-CR A_1363_ ≈ 0.065; A_898_ ≈ 0.066; LOI = 0.984;

MS-CR-Mn^2+^: A_1363_ ≈ 0.063; A_898_ ≈ 0.064; LOI = 0.984;

The fact that the LOI remains practically constant (~0.984) in all three states mathematically demonstrates that pollutant biosorption is a strictly surface phenomenon [76,77].

CR molecules and Mn^2+^ do not penetrate inside the cellulose microfibrils to destroy the lateral crystalline order of the biosorbent.

#### 3.6.15. Hydrogen Bond Intensity Index (HBI)

HBI is an indirect indicator of the degree of intermolecular interaction and organization of bound water in the matrix. It is calculated as the ratio between the hydroxyl group band (~3335 cm^−1^) and the polysaccharide backbone band (~1034 cm^−1^). HBI = A_3335_/A_1034_.

Control (MS): 0.110/0.440 = 0.250

MS-CR system: 0.082/0.225 = 0.364

MS-CR-Mn^2+^ system: 0.080/0.440 = 0.181

For the MS-CR system, the increase in HBI to 0.364 reveals the emergence of a new and dense system of intramolecular hydrogen bonds between the dye and the biomass. In contrast, the sudden decrease in the index to 0.181 in the ternary system is clear evidence of chelation and coordination of Mn^2+^ ions. The heavy metal substitutes for hydrogen atoms in the lattice, breaking the classical hydrogen bonds to form strong metal–oxygen coordination bonds (previously highlighted at 559.37 cm^−1^) [72,73,74].

#### 3.6.16. Quantitative Evaluation of Empirical Structural Indices

To convert qualitative observations into rigorous data, the relative hydrophobicity index (RHI), lateral crystallinity index (LOI), and hydrogen bonding index (HBI) were calculated, based on the mathematical absorbance ratios (Table 3) [76,77].

For a quantitative assessment of the surface modifications induced by the biosorption process, empirical structural indices (RHI, LOI and HBI) were calculated based on spectral absorbance ratios. From the point of view of hydrophobicity, the binary system shows a sharp increase in the RHI (from 0.254 to 0.390), which confirms the coverage of the hydrophilic sites of the maize stalk with the hydrophobic aromatic nuclei of the CR dye [70,75]. This dynamics is supported by the HBI index, whose variations (0.250–0.364–0.181) clearly discriminate between the physicochemical fixation of the dye through new hydrogen bonds and the chemisorption of Mn^2+^ ions [74,76].

The drastic decrease in HBI in the MS-CR-Mn^2+^ system indicates a disruption of the hydrogen bonding system as a result of the coordination of Mn^2+^ on the deprotonated active sites. Analysis of the RHI shows that CR attachment modifies the hydrophilic/hydrophobic balance of the surface by shielding the polar -OH groups and exposing the aromatic rings.

The correlation between HBI and LOI indices provides essential details regarding the localization of the process. The constancy of the lateral crystallinity index (LOI ~ 0.984) shows that the pollutant retention process does not alter the internal architecture of the plant biosorbent, being limited to superficial interactions. Finally, the absolute LOI constant ~0.984 certifies that the immobilization of pollutants does not affect the internal organization of cellulose microfibrils. On the other hand, the fluctuation of HBI (which increases to 0.364 in the MS-CR by the contribution of dye hydrogen bonds and decreases sharply to 0.181 in the MS-CR-Mn^2+^ reveals that Mn^2+^ ions are substituting hydrogen in the pattern. The adsorption of Fe^3+^ and Cr^3+^ is nevertheless confirmed by the quantitative AAS results (see Section 3.4), and by the elemental mapping obtained through SEM/EDX, which clearly shows the presence of both metals on the MS-CR surface. Additionally, Fe^3+^ and Cr^3+^ form inner-sphere complexes with oxygen-donor groups that do not always produce new, well-defined vibrational bands (see Section 3.7.2).

### 3.7. SEM/EDX Analysis

#### 3.7.1. SEM Analysis

**Figure 12:**
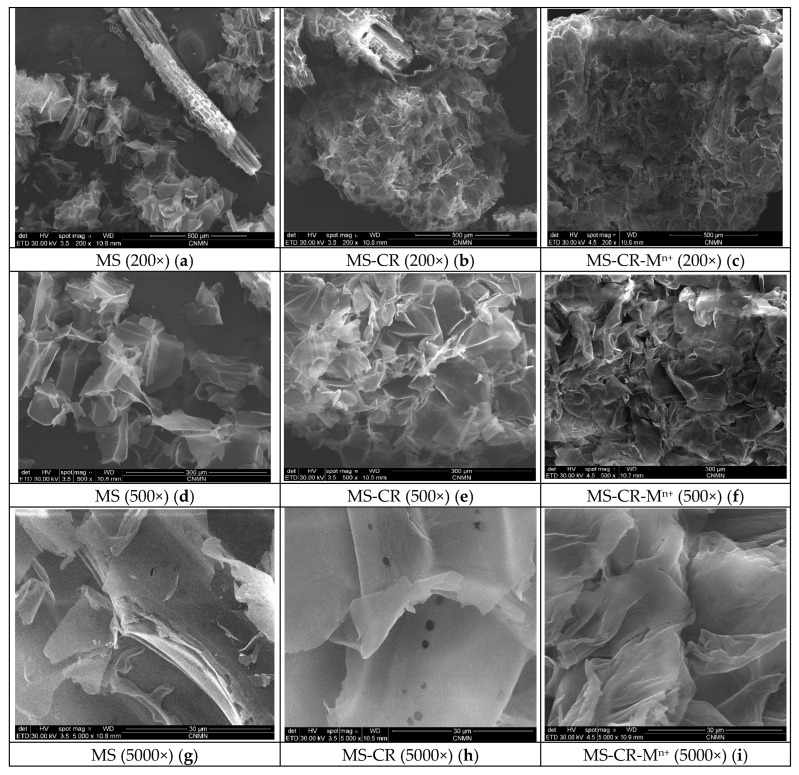
SEM image of MS (**a**,**d**,**g**); MS-CR (**b**,**e**,**h**) and MS-CR-M^n+^ (**c**,**f**,**i**).

The SEM investigation provides a clear and systematic view of the morphological evolution of the materials across the MS, the MS-CR, and the MS-CR-M^n+^ (Figure 12a–i).

The micrographs recorded at low (200×, 500×) and high magnification (5000×) reveal distinct structural transformations that validate the success of each modification step and explain the enhanced functional performance of the final material [78,79].

At low magnification (200–500×), MS exhibits a relatively homogeneous granular structure with well-defined particle boundaries and an open, accessible surface (Figure 12a,d). The particles appear loosely aggregated, with no visible surface deposits or structural collapse. High magnification images (5000×) confirm the presence of a smooth texture and clean surface domains, indicating that the support maintains its intrinsic porosity and provides a stable platform for subsequent functionalization (Figure 12g). This baseline morphology is essential, as it ensures that any structural changes observed in MS-CR and MS-CR-M^n+^ can be attributed exclusively to the chemical treatments [80].

Functionalization with CR induces a clear modification of the surface architecture.

At 200–500×, the MS-CR sample shows a more compact appearance, with particles appearing slightly more interconnected, suggesting the formation of a thin organic layer (Figure 12b,e). The surface becomes noticeably rougher, and fine deposits are visible across the material. At higher magnification, the MS-CR micrographs reveal a continuous but non-blocking coating, characterized by nanoscale irregularities uniformly distributed over the MS surface (Figure 12h). This morphology is consistent with the successful anchoring of CR molecules, which introduce additional functional groups without obstructing the pore network [81]. The MS-CR-M^n+^ sample displays the most pronounced morphological transformation. At low magnification, the surface appears denser and more heterogeneous, with visible microaggregates suggesting the presence of metal-containing domains (Figure 12c,f). High magnification SEM images (5000×) reveal the formation of granular nanoscale clusters dispersed across the surface (Figure 12i). These features are characteristic of metal–ligand coordination or deposition of M^n+^ based species. Their distribution indicates strong interactions between M^n+^ ions and the CR functional groups, confirming the efficiency of the metal loading step [82].

#### 3.7.2. EDX Analysis

EDX analysis of the MS, MS–CR, and MS–CR–M^n+^ samples provides direct and conclusive confirmation of the functionalization and adsorption process, revealing elemental changes consistent with the mechanisms proposed by FTIR and with the experimentally determined adsorption performance [83,84,85]. For native biomass (MS), the EDX spectrum is dominated by carbon (46.4% by weight) and oxygen (49.2% by weight), values characteristic of the lignocellulosic matrix rich in cellulose, hemicellulose, and lignin.

**Figure 13:**
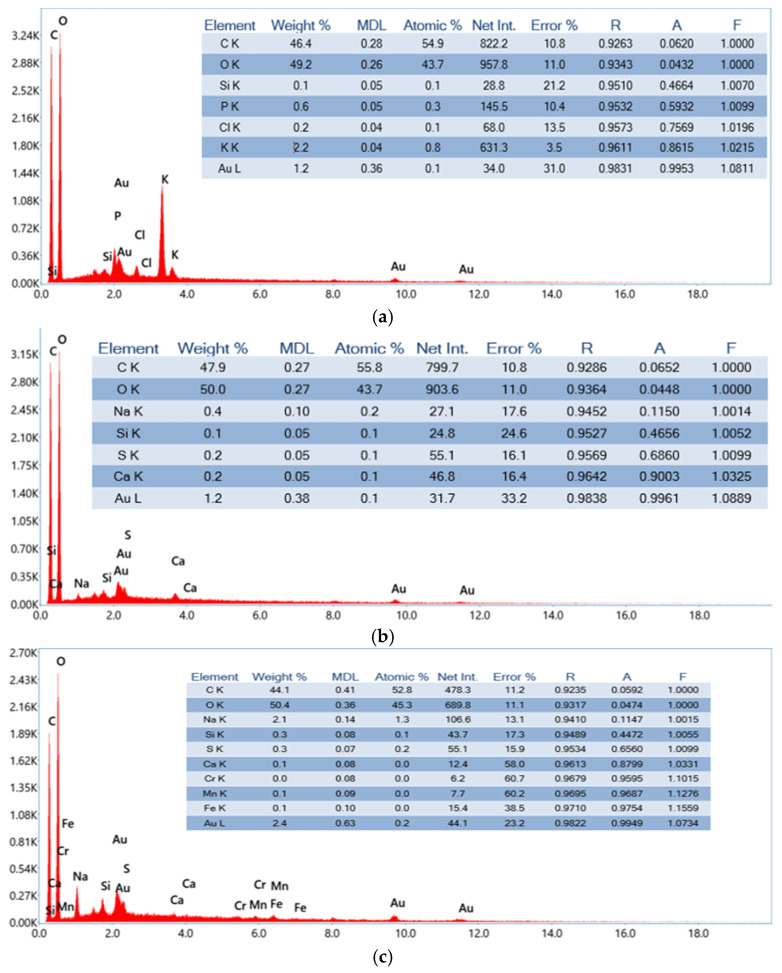
EDX spectra of MS (**a**). EDX spectra of MS-CR (**b**). EDX spectra of MS-CR-M^n+^ (**c**).

The presence of natural mineral elements—K (2.2 wt%), P (0.6 wt%), Cl (0.2 wt%), and traces of Si—reflects the intrinsic inorganic composition of the corn stalk, confirming its character as a raw biosorbent. This profile serves as a reference for evaluating changes induced by functionalization (Figure 13a).

After functionalization of MS with CR, the EDX spectrum reveals the appearance of a clear signal for sulfur (0.2 wt%), absent in the MS sample, attributable to the dye-specific sulfonate groups (–SO_3_^−^). This increase, although modest in percentage terms, represents a robust chemical marker of CR anchoring on the biomass surface.

The C and O content remains dominant, but the relative variations in intensities indicate the surface is coated with an additional organic layer, consistent with the aromatic and azo bands identified by FTIR. The presence of gold in all samples stems exclusively from the metallization required for SEM analysis (Figure 13b).

In the MS–CR–M^n+^ sample, EDX unequivocally confirms the adsorption of M^n+^, as evidenced by the appearance of characteristic signals for Mn^2+^ (0.1 wt%), Fe^3+^ (0.1 wt%), and detectable traces of Cr^3+^. Although the absolute values are small, they are significant for the initial concentrations used (2 mg/L) and for the superficial nature of the EDX technique. The net intensities of the metal lines (Mn^2+^: 7.7; Fe^3+^: 15.4; Cr^3+^: 6.2) indicate a non-uniform but clearly detectable distribution of the M^n+^ adsorbed on the functionalized surface. A notable aspect is the increase in Na content (2.1 wt%) for the solid sample loaded with M^n+^, suggesting the involvement of ion exchange between the SO_3_^−^ groups of the CR and the M^n+^, as well as possible contributions from the buffer solutions used. The coexistence of S, Na, and adsorbed M^n+^ supports the hypothesis of a mixed retention mechanism, in which electrostatic interactions, ion exchange, and metal–ligand coordination contribute simultaneously [72,73] (Figure 13c).

The mechanistic correlating EDX and FTIR data reinforces the interpretation: (i) the absence of the carboxyl band at 1243 cm^−1^ in the MS–CR–M^n+^ indicates the direct involvement of –COO^−^ groups in metal chelation; (ii) the appearance of the metal–oxygen vibration at 559.37 cm^−1^ confirms the formation of Mn–O, Fe–O, and Cr–O bonds; (iii) the intensification of the aromatic and azo bands reflects the effective coverage of the surface with CR, creating an environment rich in electron-donating sites.

Overall, the EDX analysis validates the sequence of functionalization and adsorption processes, demonstrating that MS–CR acts as a multifunctional biosorbent, capable of simultaneously retaining CR and then metal ions through a combination of hydrogen bonding, electrostatic attractions, and chelation mechanisms [67,75]. The simultaneous presence of S (CR structure) and adsorbed metals constitutes direct evidence of the efficiency of the functionalization and the critical role of the SO_3_^−^, C_6_H_5_O^−^, and COO^−^ groups in the capture of Fe^3+^, Cr^3+^, and Mn^2+^ ions. This convergence of EDX and FTIR data supports the proposed mechanistic model and explains the material’s high performance under alkaline conditions, where complete deprotonation maximizes the negative charge density and enhances metal coordination.

### 3.8. Thermal Analysis (TG/DSC) Results

**Figure 14:**
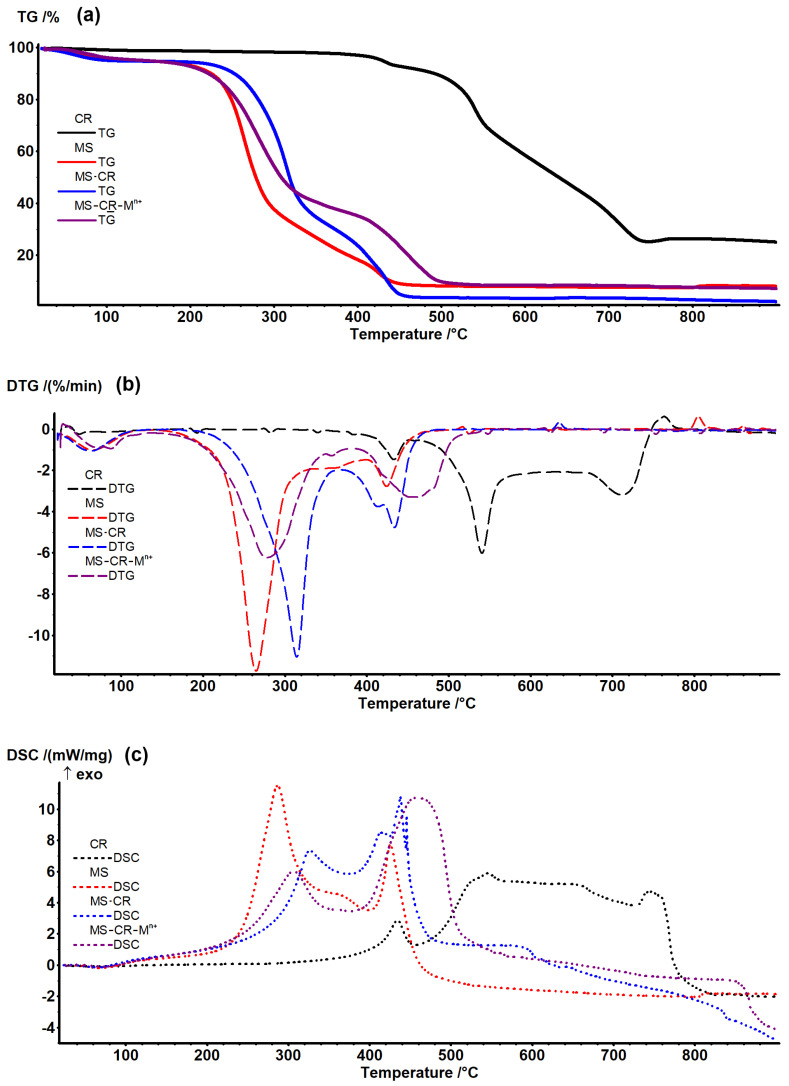
The thermogravimetric (TG) (**a**), derivative TG (DTG) (**b**) and differential scanning calorimetry (DSC) (**c**) for CR (black), MS (red), MS-CR (blue), and MS-CR-M^n+^ (purple) samples.

The individual thermal analysis results for each sample are presented in Figures S1–S4. The curves in the following help visualize the influence of the adsorbate on the thermal properties of the adsorbent: overlap of the thermogravimetric (TG) in Figure 14a, derivative TG (DTG) in Figure 14b and differential scanning calorimetry (DSC) in Figure 14c. All samples have a residual humidity, represented by water molecules that remain loosely bonded on the surface of the support. The initial mass loss assigned to the elimination of the residual solvent varies from 2.16% for CR to 5.38% for the MS-CR sample. Up to 140 °C, all MS-based samples exhibit a comparable pattern for TG, DTG and DSC curves, suggesting a similar process. By contrast the CR sample, with a mass loss that is less than half, indicates a minimum presence of humidity. Moreover, for the CR sample, the initial mass loss of 2.16% is recorded up to 380 °C, with a minor endothermic effect at 57.6 °C on the DSC curve, but with no clear step on DTG. After 380 °C the degradation of the CR organic molecule starts to unfold, firstly by cleavage of the azo bonds –N=N-, then by breaking up the aromatic rings, for a total mass loss of 72.49%, in three separate events, as indicated by the DTG peaks from 433.1, 540.8 and 711.0 °C. The presence of an exothermic peak at 434.3 °C indicates that the degradation process is accompanied by oxidation reactions. The aromatic rings are further degraded after 455 °C in a quasi-continuous way, the process being accompanied by a large, multi-peak, exothermic effect, at 545.7 and 662.0 °C, indicating the presence of multiple overlapped oxidation reactions, the broad shape suggesting the burning of carbonaceous residual mass. The residual mass consists of inorganic salt like Na_2_SO_4_ (as indicated also by the endothermic melting effect seen after 800 °C).

After 140 °C when the dehydration process is complete, the MS sample starts to degrade, the recorded mass loss being 86.89% up to 600 °C. The multiple exothermic peaks on the DSC curve, at 286.9 and 425.6 °C, indicate that overlapped oxidation reactions of the maize polysaccharides take place, the DTG peaks being observed at 265.0 and 424.2 °C [86]. The residual mass of 7.98% consists of inorganic compounds from MS.

In the case of the MS-CR sample, after elimination of moisture, the thermal degradation is retarded as indicated by all TG, DTG and DSC curves. In the temperature interval 140–600 °C, the principal degradation process takes place, with a mass loss of 91.34%. The presence of CR adsorbed by the maize granules leads to a better thermal stability, the onset of the principal mass loss event being shifted from 239.9 °C for MS to 277.9 °C for MS-CR. The improved thermal stability can also be observed on the DTG curves, the peak indicating the maximum degradation speed being shifted from 265.0 °C for the MS sample to 314.6 °C for the MS-CR sample.

The sample MS-CR-M^n+^ exhibits a smaller mass loss in the dehydration process, of only 4.34% up to 140 °C, accompanied by an endothermic effect at 71.0 °C [87] (Figure S4). The principal degradation process takes place between 140 and 600 °C, in two partially overlapped steps, with a mass loss of 86.66%. The presence of CR and metallic ions adsorbed by the maize granules has opposite influences on thermal stability, the onset of the principal mass loss event being at 239.4 °C (similar to the MS) but lower than the 277.9 °C measured for MS-CR. At the same time the DTG curve indicates that maximum degradation speed is attained at 278.8 °C, closer to the 265.0 °C peak of the MS sample than to the 314.6 °C DTG peak of the MS-CR sample.

Nevertheless, comparing the MS sample with MS-CR-M^n+^ thermal behavior, it can be observed that similar processes take place at a higher temperature for the sample containing CR and metals, the shift increasing with the temperature. The second degradation step presents a DTG peak at 453.8 °C, while for the MS sample, it occurs at 424.2 °C. The oxidation reactions responsible for the degradation steps observed on TG and DTG curves are accompanied by the strong exothermic peaks at 306.9 and 456.8 °C on the DSC curve [88].

The principal numeric data from thermal analysis are presented in Table 4 for easy comparison.

## 4. Discussion

The multi-analytical evaluation performed in this study provides convergent evidence for the functionalization of MS with CR and for the subsequent adsorption of Fe^3+^, Cr^3+^, and Mn^2+^ ions from mixed aqueous matrices. The combined use of UV–Vis quantification, adsorption experiments, FTIR-ATR, SEM/EDX, and TG-DSC/DTG enabled a coherent interpretation of the structural, chemical, and thermal modifications induced by CR immobilization and metal binding.

FTIR analysis revealed the appearance of characteristic azo-aromatic vibrations (1601–1506 cm^−1^) and a broad band at 558.50 cm^−1^ associated with deformation modes of CR and its sulfonic groups. These features, absent in native maize stalk, constitute direct vibrational markers of CR incorporation.

Although the O–H stretching band exhibited only a minor shift (3338.91 → 3335.54 cm^−1^), this effect was considered supportive rather than primary evidence. More conclusive changes—particularly the shift of the carboxyl/ester band from 1243 to 1233 cm^−1^—indicate chemical interaction between CR and the lignocellulosic matrix.

EDX analysis confirmed functionalization by detecting sulfur and sodium originating from CR, elements not present in native MS. UV–Vis quantification demonstrated CR loading (41.4–48.0 mg/g) and negligible desorption under 2 M HCl/NaOH, confirming the strong anchoring of the CR and the stability of the functional layer.

CR was selected as a functionalizing agent due to its rich chemical functionality (azo groups, sulfonic groups, aromatic rings), which introduces multiple electron-donor sites capable of coordinating multivalent metal ions. Functionalization increases both the density and diversity of active sites, as reflected in the enhanced thermal stability (TG-DSC) and the presence of S and Na in EDX spectra. Literature reports present [89,90,91,92,93,94,95,96,97,98,99] similarly highlight the suitability of CR for modifying biomass surfaces and improving adsorption performance. Moreover, the distinct vibrational bands of CR facilitate spectroscopic assessment of functionalization and metal binding, making it particularly advantageous for mechanistic studies.

The adsorption of Fe^3+^, Cr^3+^, and Mn^2+^ onto MS–CR was elucidated through the combined interpretation of FTIR, EDX, thermal analysis, and adsorption capacity measurements. The disappearance of the 1233 cm^−1^ band in the MS–CR–Mn^2+^ spectrum indicates the selective involvement of carboxyl groups in metal coordination, while the emergence of a metal–oxygen vibration near 559.37 cm^−1^ confirms the formation of coordination bonds between CR-modified sites and metal ions.

EDX spectra corroborated these findings by detecting Fe, Cr, and Mn on the MS–CR surface, demonstrating that the functional groups introduced by CR act as effective binding sites. The agreement between vibrational and elemental analyses supports a mechanism involving electrostatic attraction, chelation through –COO^−^ groups, and secondary interactions with azo-aromatic domains.

These mechanistic features are consistent with the high adsorption capacities obtained experimentally: Fe^3+^: 2.00 mg/g; Cr^3+^: 1.64 mg/g; and Mn^2+^: 1.46 mg/g.

The relative order Fe^3+^ > Cr^3+^ > Mn^2+^ reflects differences in charge density and ligand affinity. The higher uptake of Fe^3+^ and Cr^3+^ is consistent with their stronger Lewis acidity and greater propensity to form inner-sphere complexes with deprotonated carboxyl and sulfonate groups. Mn^2+^, although divalent, exhibits high removal efficiency at alkaline pH due to the fully deprotonated MS-CR surface and the dominance of electrostatic attraction.

TG-DSC and DTG analyses provided additional insight into the structural stabilization induced by CR and metal adsorption. The main degradation step shifted from 239.9 °C (MS) to 277.9 °C (MS–CR), indicating enhanced thermal stability due to CR incorporation. The DTG peak corresponding to maximum degradation rate shifted from 265.0 °C (MS) to 314.6 °C (MS–CR), further confirming the stabilizing effect.

In MS–CR–M^n+^, the onset of degradation approached that of native MS, reflecting the combined influence of CR and metal ions. However, the second major degradation event shifted from 424.2 °C (MS) to 453.8 °C (MS–CR–M^n+^), suggesting that metal coordination introduces additional stabilization at higher temperatures. These thermal signatures align with FTIR and EDX observations and support the adsorption capacities measured experimentally.

## 5. Conclusions

The results obtained in this study demonstrate that functionalizing MS with CR produces a robust biosorbent capable of effectively retaining multivalent metal ions under various environmental conditions.

The functionalization at pH 10 maximizes the CR loading (≈48 mg/g), confirming that the complete deprotonation of the –OH and –COOH groups increases pore accessibility and favors hydrophobic and π–π interactions.

The high stability of CR on MS, even in 2 M HCl/NaOH, demonstrates that the binding is not only electrostatic but involves extensive networks of hydrogen bonds and aromatic binding within the lignin, which justifies the use of the material in industrially aggressive environments. The absence of detectable CR desorption in extreme environments confirms that the material poses no risk of secondary contamination, a major advantage for biosorbents modified with anionic dyes.

The consistent increase in retention from pH 2 to 10 confirms that the progressive deprotonation of the –SO_3_^−^, –O^−^, and –COO^−^ groups is critical for the formation of metal–ligand complexes.

The results confirm that MS-CR can be used in multi-metal treatments, with selectivity varying depending on pH. The increase of Q_e_ up to 2.00 mg/g Fe^3+^, 1.64 mg/g Cr^3+^, and 1.46 mg/g Mn^2+^demonstrates retention via electrostatic interactions as well as complexation mechanisms, even at high concentrations.

The decrease in R (%) at high concentrations confirms the gradual saturation of the adsorption sites, but the continued increase in Q_e_ indicates metal adsorption as a function of the concentration gradient.

Desorption with 0.5 M HCl is optimal because it ensures complete protonation of the active sites without degrading the matrix. Desorption rates of 90–97% demonstrate that the material is regenerable, which reduces operational costs and allows for use in multiple cycles.

The investigation by FTIR analysis of MS, MS-CR and MS-CR-Mn^2+^ processed form, allowed the elucidation of the intermolecular mechanisms that govern the processes of concomitant adsorption of CR and metal ions.

FTIR data demonstrate that MS functions as a multifunctional surface biosorbent. Pollutant retention combines two independent and simultaneous mechanisms.

The mechanism of retention of the organic CR: Comparative analysis of the spectra revealed a hypochromic shift of the broad band associated with hydroxyl groups from 3338.91 to 3335.54 cm^−1^, accompanied by a decrease in its intensity. This spectroscopic behavior demonstrates the active involvement of the -OH groups of cellulose and hemicellulose in establishing strong hydrogen bonds with the functional groups of the CR. Additionally, the appearance and intensification of bands in the 1601–1506 cm^−1^ area show the grafting of the aromatic skeleton and azo bonds (-N=N-) of CR on the MS matrix.

Mechanism of adsorption of Mn^2+^: Spectral analysis reveals a distinct role of carboxylic groups in the MS mass. The band at 1243.60 cm^−1^ (specific to ester/carboxylic bonds in hemicellulose), although partially modified upon CR adsorption (1233.26 cm^−1^), undergoes complete extinction in the complexing system with Mn^2+^. This structural transformation indicates local deprotonation and direct participation of carboxylic oxygen in the chemisorption process through the formation of coordination complexes with Mn^2+^ ions. The hypothesis is experimentally confirmed by the major spectral change in the low fingerprint area, where the band at 526.65 cm^−1^ shifts significantly to 559.37 cm^−1^ in the presence of Mn^2+^, a band characteristic of stretching vibrations of newly formed metal–oxygen (Mn-O) bonds.

Structural integrity of the MS: From a morpho-structural point of view, the maintenance of the unchanged position of the major peak at 1034 cm^−1^ (vibration of the C-O-C polysaccharide skeleton of cellulose) throughout all treatments demonstrates that the processes of biosorption and retention of pollutants are non-destructive. The phenomenon is limited to surface phenomena (physical adsorption and electrostatic chemisorption/chelation), without altering the internal crystalline network of the biomass in the MS. So, MS behaves as an efficient multifunctional biosorbent, capable of retaining CR through hydrogen bonds and aromatic interactions, simultaneously providing specific carboxylic and chelating sites for the immobilization of Mn^2+^.

The structural stability observed via TG-DSC indicates an increase in the degradation temperature, suggesting a genuine chemical interaction between the CR and the lignocellulosic matrix, rather than merely surface adsorption.

SEM highlights increased roughness and the appearance of metal clusters, while EDX confirms the presence of S, Na, and adsorbed metals, supporting mixed mechanisms: electrostatic, ion exchange, and coordination.

While the maximum capacities are below 2 mg/g, they fall within the expected range for lignocellulosic biosorbents functionalized with sulfonated azo dyes. The strength of MS–CR lies not in achieving extreme Q_e_ values, but in its unique combination of structural stability, selective coordination, high removal efficiencies at low concentrations, and fully green, scalable synthesis—features that are essential for practical deployment in low-cost water treatment systems.

Thus, following the adsorption/desorption studies, it was concluded that MS-CR is a scalable, biodegradable, and economical adsorbent, suitable for treating water with low–moderate concentrations of metals. At the same time, its stability in extreme environments makes it suitable for real-world industrial effluents, not just laboratory conditions. And its high regeneration capacity positions it as a sustainable alternative to conventional materials.

## Figures and Tables

**Table 1:** Peaks observed at different wavelengths (cm^−1^) in the FTIR spectrum and functional group assignments corresponding to molecular vibrations.

Wavenumber (cm^−1^)	Possible Assignment	Chemical Meaning
3335	O–H stretching	Hydroxyl groups (alcohol or phenol from cellulose, lignin, or absorbed water)
2899	C–H stretching	Aliphatic C–H bonds, from –CH_2_/–CH_3_ groups in cellulose and hemicellulose
1601	C=C or C=O stretching	Aromatic ring vibration (lignin) or amide/C=O from CR
1509	Aromatic C=C stretching	Typical of aromatic rings (lignin or azo CR)
1333	C–N stretching or O–H bending	May arise from CR amine groups or cellulose
1233	C–O–C stretching	Ether linkage in cellulose or hemicellulose
1106	C–O stretching	Polysaccharide (cellulose/hemicellulose backbone)
1034	C–O–C or C–O–H vibrations	Common strong band of polysaccharides (cellulose)
898	β-glycosidic linkage	Characteristic of cellulose structure
832–650	Aromatic out-of-plane bending	Indicative of substituted benzene rings—common in CR
585, 438	Skeletal bending or metal–oxygen vibrations	Possibly weak lattice or inorganic vibrations

**Table 2:** Assignment of FTIR bands and comparative analysis of spectral changes in the adsorption process.

Chemical Assignment of Molecular Bands	MS (cm^−1^)	MS-CR (cm^−1^)	MS-CR-Mn^2+^ (cm^−1)^	Interaction Type/Mechanism Identified
Stretching vibration -OH and –NH (cellulose/CR)	3338.91	3335.54	3335.96	Formation of hydrogen bonds between biomass and -NH groups
Aliphatic stretching vibration C-H (-CH_2_, -CH_3_)	2898.10	2899.38	2898.09	Stable aliphatic skeleton; minimal network changes.
Aromatic rings C=C/ Azo-N=N- bonds	1602.78	1601.88	1595.16	Adsorption of the aromatic skeleton; hydrophobic interaction of type π-π.
Secondary vibration of aromatic ring	—	1506.99	1506.66	Shows the grafted aromatic structure on the biomass surface
C-H strain (cellulose matrix)	1365.00	1363.16	1363.13	Structural stability of the main polysaccharide components.
C-) stretch ester/carboxylic groups	1243.60	1233.26	Missing/Masked	Deprotonation of C=O groups in hemicellulose/contribution to metal chelation
Asymmetric stretching C-O-C (Ether bonds)	1160.03	1160.63	1160.28	Decrease in relative intensity due to surface shielding by pollutants.
C-O and C-C stretch (major peak in cellulose)	1034.89	1034.56	1034.83	Structural stability marker; adsorption strictly at the biosorbent surface
Low-strain vibrations/metal–oxygen bonds	526.65	558.50	559.37	Evidence of Mn^2+^ chemisorption through the formation of Mn-O coordination bonds

**Table 3:** Dynamics of FTIR structural parameters of MS, MS-CR and MS-CR-Mn^2+^.

Experimental System	Relative Hydrophobicity Index (RHI) [A_2898_/A_3335_]	Lateral Crystallinity Index (LOI) [A_1363_/A_898_]	Hydrogen Bonding Index (HBI) [A_3335_/A_1034_]
MS	0.254	0.983	0.250
MS-CR	0.390	0.984	0.364
MS-CR-Mn^2+^	0.362	0.984	0.181

**Table 4:** Principal numeric data from thermal analysis for CR, MS, MS-CR and MS-CR-M^n+^ samples.

Sample	Humidity/Temperature	Endothermic Peak	Degradation	Exothermic Peaks	Residual Mass
CR	2.16%/380 °C	57.6 °C	72.49%	434.3/545.7/662.0 °C	24.97%
MS	5.25%/140 °C	68.9 °C	86.89%	286.9/425.6 °C	7.98%
MS-CR	5.38%/140 °C	63.6 °C	91.34%	326.5/417.0/438.1 °C	2.05%
MS-CR-M^n+^	4.34%/140 °C	71.0 °C	86.66%	306.9/456.8 °C	7.11%

## Data Availability

The original contributions presented in this study are included in the article. Further inquiries can be directed to the corresponding author.

## Supplementary Materials

The following supporting information can be downloaded at https://www.mdpi.com/article/10.3390/polym18131600/s1. Figure S1. TG (black), DSC (dotted blue) and DTG (dashed green) curves for the CR sample; Figure S2. TG (black), DSC (dotted blue) and DTG (dashed green) curves for the MS sample; Figure S3. TG (black), DSC (dotted blue) and DTG (dashed green) curves for the MS-CR sample; and Figure S4. TG (black), DSC (dotted blue) and DTG (dashed green) curves for the MS-CR-Mn+ sample.
