# Supplementary material for: Congo Red–Functionalized Maize Stalk for Fe3+, Cr3+ and Mn2+ Adsorption: Multi-Analytical Characterization of Interaction Mechanisms
Source: Polymers (Basel). 2026 Jun 27;18(13):1600. doi: 10.3390/polym18131600 (PMC13364213; doi:10.3390/polym18131600)
Supplement: Supplementary file 1 [file polymers-18-01600-s001.zip › polymers-4379195-supplementary.pdf]

polymers

MDPI

Article

Congo Red–Functionalized Maize Stalk for Fe^{3+}, Cr^{3+} and Mn^{2+} Adsorption: Multi-Analytical Characterization of Interaction Mechanisms

Nicoleta Mirela Marin [1,2,3,*], Toma Galaon [1,2], Adriana Mariana Borş [4], Roxana Doina Trusca [5], Ludmila Motelica [5,6] and Ovidiu Oprea [5,7,8]

1 National Research and Development Institute for Industrial Ecology ECOIND, Street Podu Dambovitei No. 57-73, District 6, 060652 Bucharest, Romania; tomagalaon@yahoo.com
2 Department of Analytical and Physical Chemistry, University of Bucharest, 4-12 Regina Elisabeta Bd., 030018 Bucharest, Romania
3 Department of Oxide Materials Science and Engineering, National University of Science and Technology POLITEHNICA Bucharest, 1–7 Gh. Polizu, 060042 Bucharest, Romania
4 National Institute for R&D for Optoelectronics-Subsidiary, Research Institute for Hydraulics and Pneumatics—INOE 2000-IHP, 040558 Bucharest, Romania; bors.ihp@fluidas.ro
5 National Centre for Micro- and Nanomaterials, National University of Science and Technology POLITEHNICA Bucharest, 313 Independence Boulevard, 060042 Bucharest, Romania; truscaroxana@yahoo.com (R.D.T.); ludmila.motelica@upb.ro (L.M.); ovidiu.oprea@upb.ro (O.O.)
6 Research Center for Advanced Materials, Products and Processes, National University of Science and Technology POLITEHNICA Bucharest, Splaiul Independenței 313, 060042 Bucharest, Romania
7 Academy of Romanian Scientists, 3 Ilfov St., 050045 Bucharest, Romania
8 Faculty of Chemical Engineering and Biotechnologies, National University of Science and Technology POLITEHNICA Bucharest, Gh. Polizu 1-7, 011061 Bucharest, Romania
* Correspondence: nicoleta.marin@incdecoind.ro

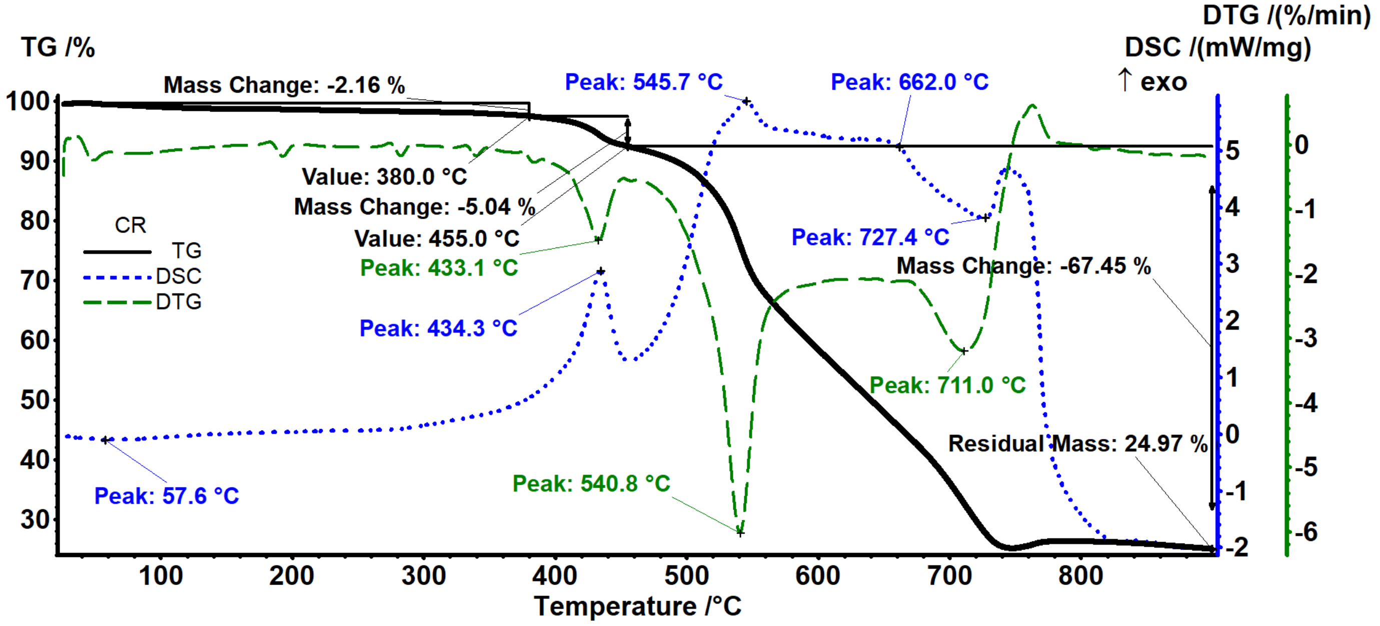

Figure S1. TG (black), DSC (dotted blue) and DTG (dashed green) curves for the CR sample

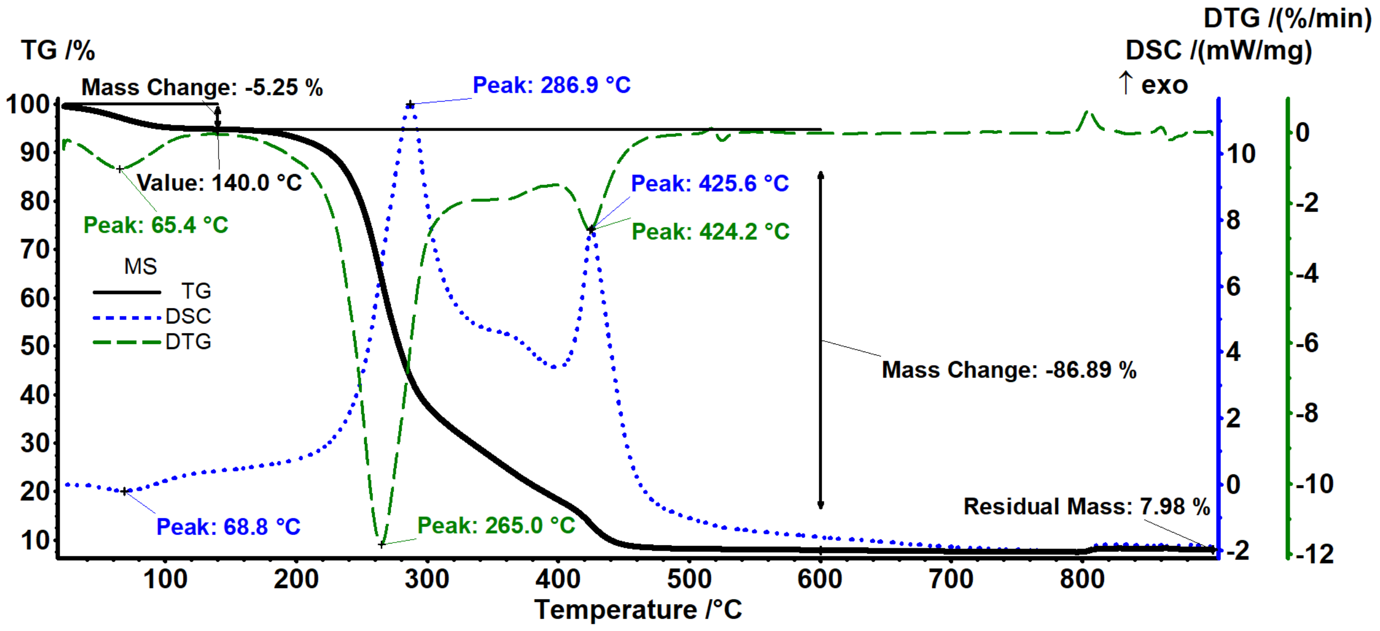

Figure S2. TG (black), DSC (dotted blue) and DTG (dashed green) curves for the MS sample

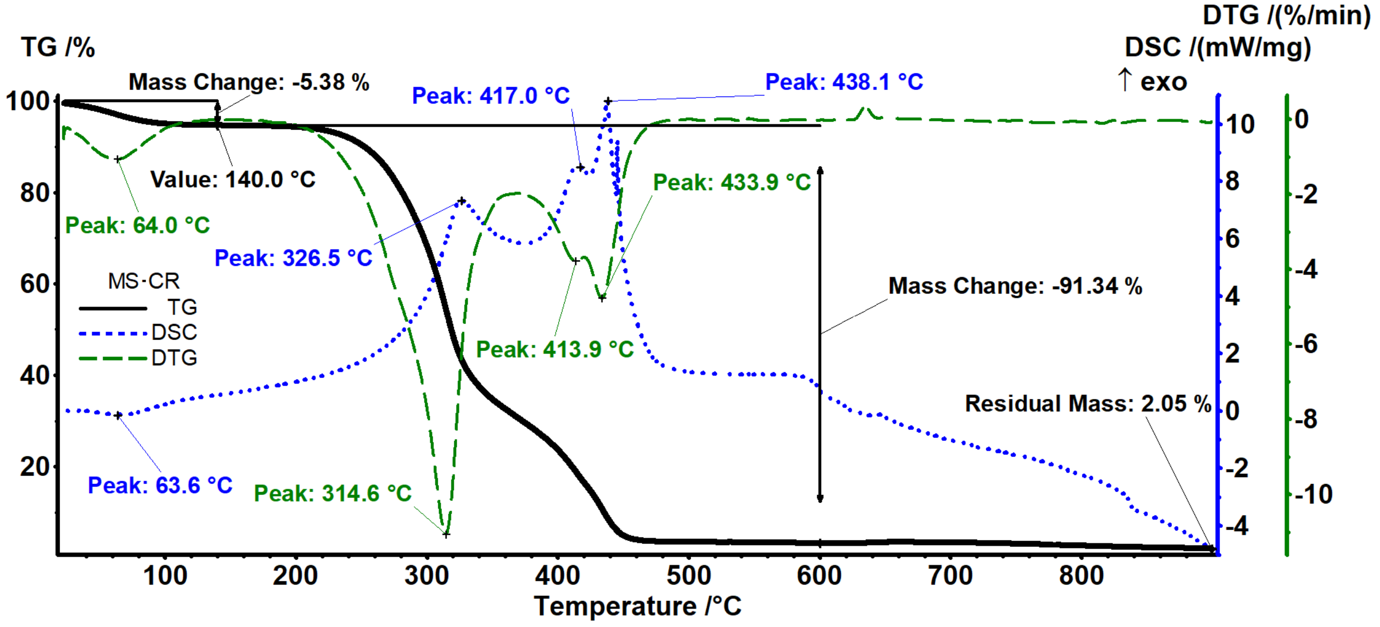

Figure S3. TG (black), DSC (dotted blue) and DTG (dashed green) curves for the MS-CR sample

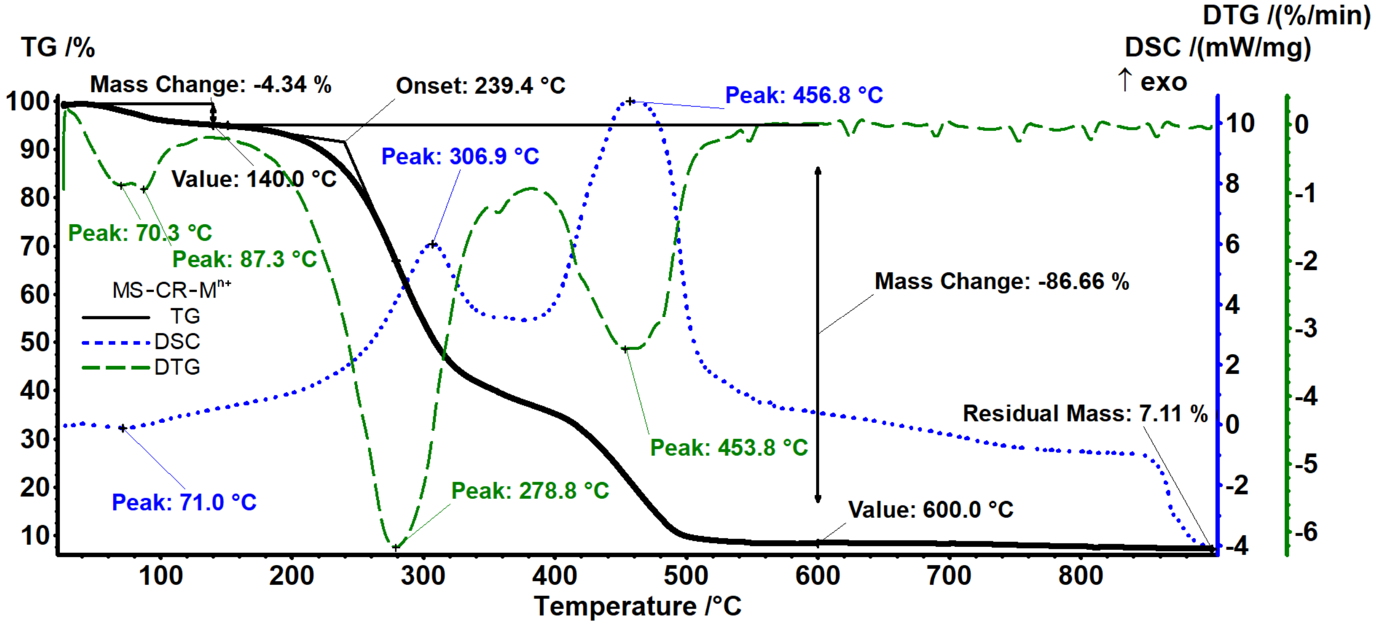

Figure S4. TG (black), DSC (dotted blue) and DTG (dashed green) curves for the MS-CR-M^{n+} sample
